# The Tumor-Associated Variant RAD51 G151D Induces a Hyper-Recombination Phenotype

**DOI:** 10.1371/journal.pgen.1006208

**Published:** 2016-08-11

**Authors:** Carolyn G. Marsden, Ryan B. Jensen, Jennifer Zagelbaum, Eli Rothenberg, Scott W. Morrical, Susan S. Wallace, Joann B. Sweasy

**Affiliations:** 1 Department of Microbiology and Molecular Genetics, The Markey Center for Molecular Genetics, University of Vermont, Burlington, Vermont, United States of America; 2 Department of Therapeutic Radiology, Yale University School of Medicine, New Haven, Connecticut, United States of America; 3 Department of Department of Biochemistry and Molecular Pharmacology, NYU School of Medicine, New York, New York, United States of America; 4 Department of Biochemistry, University of Vermont, Burlington, Vermont, United States of America; University of Washington School of Medicine, UNITED STATES

## Abstract

The RAD51 protein plays a key role in the homology-directed repair of DNA double-strand breaks and is important for maintaining genome stability. Here we report on a novel human RAD51 variant found in an aggressive and therapy-refractive breast carcinoma. Expression of the RAD51 G151D variant in human breast epithelial cells increases the levels of homology-directed repair. Expression of RAD51 G151D in cells also promotes high levels of chromosomal aberrations and sister chromatid exchanges. *In vitro*, the purified RAD51 G151D protein directly and significantly enhances DNA strand exchange activity in the presence of RPA. In concordance with this result, co-incubation of G151D with BRCA2 resulted in a much higher level of strand-exchange activity compared to WT RAD51. Strikingly, the RAD51 G151D variant confers resistance to multiple DNA damaging agents, including ionizing radiation, mitomycin C, and doxorubicin. Our findings demonstrate that the RAD51 G151D somatic variant has a novel hyper-recombination phenotype and suggest that this property of the protein is important for the repair of DNA damage, leading to drug resistance.

## Author Summary

Therapeutic resistance is a major hurdle for the treatment and eradication of cancer. Furthermore, the development of therapeutic resistance significantly decreases patient survival and negatively impacts the quality of life of patients battling cancer. Cancer cells utilize a number of previously described mechanisms in order to overcome sensitivity to cancer therapeutics, including overexpression of RAD51. However, in this study we report a novel gain-of-function heterozygous somatic variant, RAD51 G151D, identified in a highly refractory and aggressive breast adenocarcinoma. RAD51 G151D induces a hyper-recombination phenotype in human cells resulting in increased resistance to therapeutics via enhanced HDR of DSBs. We further demonstrate enhanced DNA strand exchange activity in the presence of RPA, providing a possible mechanism for the hyper-recombination phenotype observed in cells. Our study presents a novel hyper-recombinant RAD51 tumor-associated variant (RAD51 G151D), providing the first evidence that links altered RAD51 function with therapeutic resistance as well as a novel genetic marker to identify patients at high risk for aggressive and refractory disease.

## Introduction

Human RAD51 is a RecA-like recombinase required for HDR (homology-directed repair) of DSBs (double-strand breaks), forming helical nucleoprotein filaments on DNA in an ATP-dependent manner and catalyzing strand exchange between homologous sequences. RAD51 is an essential protein for genome maintenance with roles in both HDR and replication fork maintenance [1–4]. Both germline and somatic mutations in HDR genes are clearly involved in the initiation and progression of cancer [5–12]. Highlighting this link between HDR and carcinogenesis is the incredibly high lifetime risk for cancer sustained by carriers of BRCA1 and BRCA2 mutations [13–18]. Since RAD51 is a vital component of HDR, it has been hypothesized that missense mutations that significantly alter its function or regulation would be highly deleterious and therefore likely not tolerated in cells. However, there is a dearth of data reporting the cellular effects of cancer-associated RAD51 variants.

Currently, the association between cancer incidence and/or progression and RAD51 is strictly correlative. Previous studies have shown elevated RAD51 expression levels in prostate cancer, invasive breast cancer and small cell lung cancers [19–22]; however, decreased RAD51 expression levels in breast tumors and breast cancer cell lines have also been reported [23]. Naturally occurring single nucleotide polymorphisms (SNPs) of RAD51 have been identified in the population in association with cancer. RAD51 G135C is a naturally occurring variant in the 5’ untranslated region of RAD51 that was shown to increase breast cancer incidence in BRCA2 mutation carriers and gastric cancer. Mutation of G to C at position 135 increases the promoter activity of RAD51 thereby elevating RAD51 expression levels, one possible mechanism underpinning the contribution of this mutation to cancer susceptibility. Nonetheless, additional studies are needed to elucidate the link between RAD51 G135C and cancer etiology. Another germline variant, RAD51 R150Q, was identified in a study conducted in Japanese hereditary breast cancer patients [24], yet association with disease incidence was not definitive. More recently, a dominant-negative RAD51 mutation, T131P, was identified in a Fanconi anemia-like patient [25]. Cells expressing RAD51 T131P were found to be proficient in HR but defective in DNA interstrand cross-link repair (ICL) [25]. These data, in combination with other studies, exemplify the function of RAD51 during DNA replication and maintenance of replication fork stability in addition to its established function in HDR of DSBs [25–29]. Clearly, proper RAD51 function is important for multiple cellular processes and crucial for maintaining genome stability. Therefore, RAD51 SNPs identified in the general population may yield clues to better understand both RAD51 function and how dysfunction may lead to an increased risk of cancer and/or acquired resistance to standard of care treatments.

In this study, we investigated the cellular effects of a heterozygous somatic tumor variant, RAD51 G151D, we identified in 1 out 32 breast tumors analyzed by DNA sequencing. Previously, we demonstrated that the RAD51 G151D protein possesses altered biochemical and biophysical properties [30]. Located on the outer surface, the G151D mutation confers a net electronegative charge propagated throughout the RAD51 filament likely affecting biomechanical properties and protein-protein interactions [30]. In fact, the G151D mutation appears to decrease filament stiffness, which, in combination with the altered surface charge, dramatically increases the electrophoretic mobility of both RAD51-ssDNA and RAD51-dsDNA filaments [30]. Significantly, RAD51 G151D forms mixed filaments with WT RAD51 on DNA and exhibits intermediate physical properties [30]. These findings suggest mixed filament formation is possible both within the heterozygous patient cells as well as in our human cell models. Collectively, the data indicate that mixed filaments are likely to give rise to profound biochemical and biological phenotypes.

RAD51 G151D was identified in an African-American woman with early-onset infiltrating ductal adenocarcinoma. After failed radiation and chemotherapy treatment at the primary site, the patient developed metastatic disease only a year after the initial diagnosis. Attempts to eradicate the sites of metastasis using radiation and chemotherapy were unsuccessful. Given the potential linkage of RAD51 with chemo-resistance and genome instability [31–35], we proposed that the refractory and aggressive characteristic of the tumor was induced by expression of the RAD51 G151D variant. We provide evidence that expression of RAD51 G151D in both non-transformed and transformed human cells results in a hyper-recombination phenotype leading to increased HDR of DSBs and increased resistance to DNA damaging agents. We also demonstrate that the RAD51 G151D protein itself possesses enhanced DNA strand exchange activity, possibly uncovering novel regulatory mechanisms of RAD51.

## Results

### Exogenously expressed RAD51 G151D increases homology-directed repair of double-strand DNA breaks

**Fig 1:**
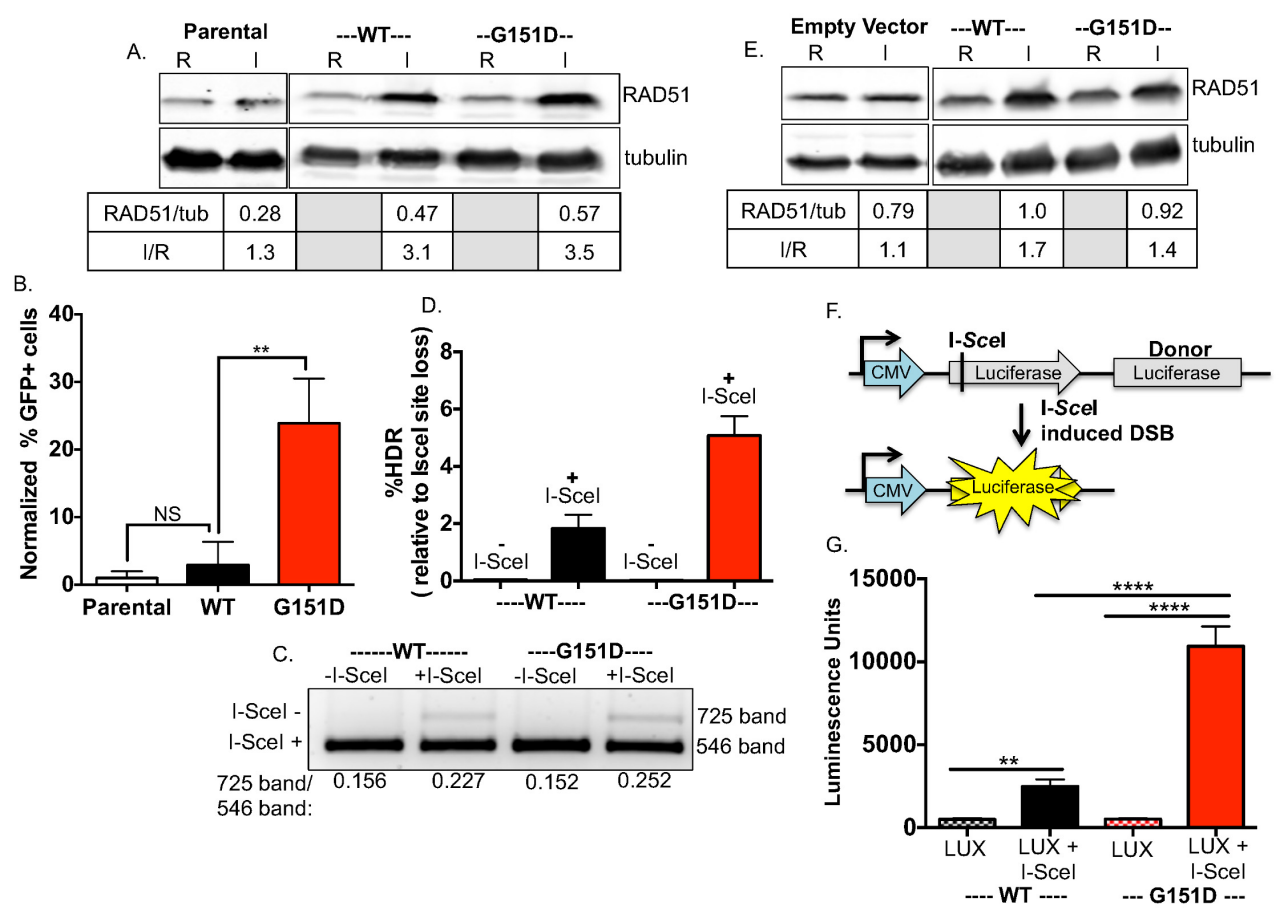
Enhanced HDR of chromosomal DSBs in cell lines expressing RAD51 G151D. **A.** RAD51 WT and G151D were stably expressed in MCF7 cells harboring the I-*Sce*I reporter construct using the pRVY TET-OFF inducible expression vector. The addition of doxycycline to the media turns off exogenous RAD51 expression (repressed, abbreviated R; endogenous RAD51 protein levels only), with expression induced upon removal of DOX (induced, abbreviated I; endogenous levels + exogenous protein levels). Western blot with an antisera raised against RAD51 protein demonstrates equivalent expression of exogenous WT and G151D (I) in their respective MCF-7 DR-GFP pools (RAD51/tubulin), as well as the fold increase in expression over endogenous RAD51 (I/R). **B.** The percentage of GFP positive cells was measured by flow cytometry 72hrs after nucleofection with an I-*Sce*I expression vector. The percentage of GFP-positive cells from MCF-7 DR-GFP parental cells was normalized to 1 and the relative change of percent GFP-positive cells from MCF-7 DR-GFP RAD51 WT and G151D cells was calculated. Data are graphed as mean ± SD from 3 independent experiments ** p<0.01. **C,D.** Analysis of I-*Sce*I site loss **(C)** and percent HDR relative to I-*Sce*I site loss **(D).** PCR amplification of the DR-GFP cassette was performed using genomic DNA as a template isolated from RAD51 WT or RAD51 G151D expressing MCF-7 DR-GFP cells nucleofected without DNA or with the I-*Sce*I expression vector. **C.** To determine the percent I-*Sce*I site loss, the 725bp and 546bp bands from each lane were quantified using Quantity One software. Representative agarose gel demonstrating the digested PCR products. I-*Sce*I (-) labels the 725bp band indicative of I-*Sce*I site loss; I-*Sce*I (+) labels the 546bp indicative of maintenance of the I-*Sce*I site. **D.** The percent HDR was calculated by dividing the percent GFP+ cells by the percent I-*Sce*I site loss after nucleofection of the I-*Sce*I expression vector or no DNA. The graphed percentages are the mean ± SD of 3 independent experiments. **E.** Western blot demonstrates equivalent expression of exogenous WT and G151D (I) in their respective MCF10A pools (RAD51/tubulin), as well as the fold increase in expression over endogenous RAD51 (I/R). **F.** Schematic of the HDR luciferase reporter assay. The I-*Sce*I recognition site is located within the luciferase ORF, disrupting luciferase expression. A luciferase donor DNA (lacking a promoter) is located downstream as a template for HDR. Co-expression of the I-SceI endonuclease and luciferase reporter results in DSB formation in the luciferase reporter construct. **G.** The I-*Sce*I luciferase reporter (LUX) and I-*Sce*I nuclease (I-SceI) were nucleofected into MCF10A RAD51 WT or G151D expressing cells. Cell lysates were collected 24 hours post-transfection and assayed for luciferase activity. Relative luminescent units for RAD51 WT or G151D expressing MCF10A cells nucleofected with the I-*Sce*I luciferase reporter (LUX) alone (control for background levels) or in combination with I-*Sce*I nuclease (I-SceI) are graphed. Error bars are SEM (n = 2).

To determine the HDR efficiency of RAD51 G151D activity at DSBs, we generated MCF-7 DR-GFP cells with stable and equivalent expression of RAD51 WT or G151D (Fig 1A) and performed the DR-GFP reporter assay [36]. In this assay, expression of the rare-cutting endonuclease I-*Sce*I results in a chromosomal DSB at an integrated *I-SceI* recognition site in a gene encoding for GFP (*SceGFP*). Repair of the I-*Sce*I-induced double strand break via HDR, using a truncated GFP repeat (*iGFP*) downstream as a template, results in restoration of a functional GFP gene subsequently measured by flow cytometry. As shown in Fig 1B, expression of RAD51 G151D results in a significant increase in GFP positive cells compared to either exogenously expressed RAD51 WT or the parental MCF-7 DR-GFP cells. Therefore, expression of RAD51 G151D, but not RAD51 WT expression, results in a significant increase in HDR of an I-*Sce*I-induced DNA DSB.

Although HDR of an I*-Sce*I-induced DNA DSB restores GFP expression, repair by other pathways such as non-homologous end-joining (NHEJ) or single-strand annealing (SSA) can occur. Repair by HDR, NHEJ or SSA each result in loss of the I-*Sce*I recognition site, therefore I-*Sce*I site loss can be used to query overall DSB repair [36]. RAD51 G151D expressing cells exhibited slightly higher levels of I-*Sce*I site loss compared to RAD51 WT expressing cells (Fig 1C), supporting the hyper-recombinant activity of RAD51 G151D. The fairly equivalent levels of I-*Sce*I site loss in RAD51 WT and G151D expressing cells indicate a lack of significant effect of RAD51 G151D on DSB repair by non-homologous end joining (NHEJ) and single-strand annealing (SSA). However, dividing the percentage of GFP positive cells by the percentage of I-*Sce*I site loss demonstrates increased HDR in RAD51 G151D expressing cells as compared to WT expressing cells (Fig 1D) [36]. We confirmed increased HDR levels associated with G151D expression in another human cell line, MCF10A, an immortalized non-transformed breast epithelial cell line (Fig 1E) using a luciferase-based HDR assay (schematic in Fig 1F) [37]. Similar to the DR-GFP assay, an I-*Sce*I site has been integrated into the open reading frame of a coding sequence for luciferase thereby disrupting expression. Downstream is another coding sequence for luciferase that lacks a promoter, and therefore expression is prevented. Repair of an I-*Sce*I induced DSB by HDR using the promoter-less luciferase sequence downstream as a template for repair restores luciferase expression. MCF10A cells expressing RAD51 WT or G151D were nucleofected with no DNA (negative control), the luciferase reporter construct alone (labeled LUX) (control for background), or the luciferase reporter construct and an I-*Sce*I construct (labeled I-SceI). Restoration of luciferase expression was measured by the production of light upon catalysis of the substrate luciferin. As shown in Fig 1G, MCF10A cells expressing RAD51 G151D demonstrated a significant increase in luciferase expression (measured by luminescence units) as compared to WT expressing cells, indicative of a significant increase in HDR of the I-*Sce*I induced DSB. Collectively, these data demonstrate expression of RAD51 G151D significantly increases HDR of a nuclease-induced DNA DSB suggesting that it confers a hyper-recombination phenotype.

### Exogenously expressed RAD51 G151D increases spontaneous sister chromatid exchanges and RAD51 foci

**Fig 2:**
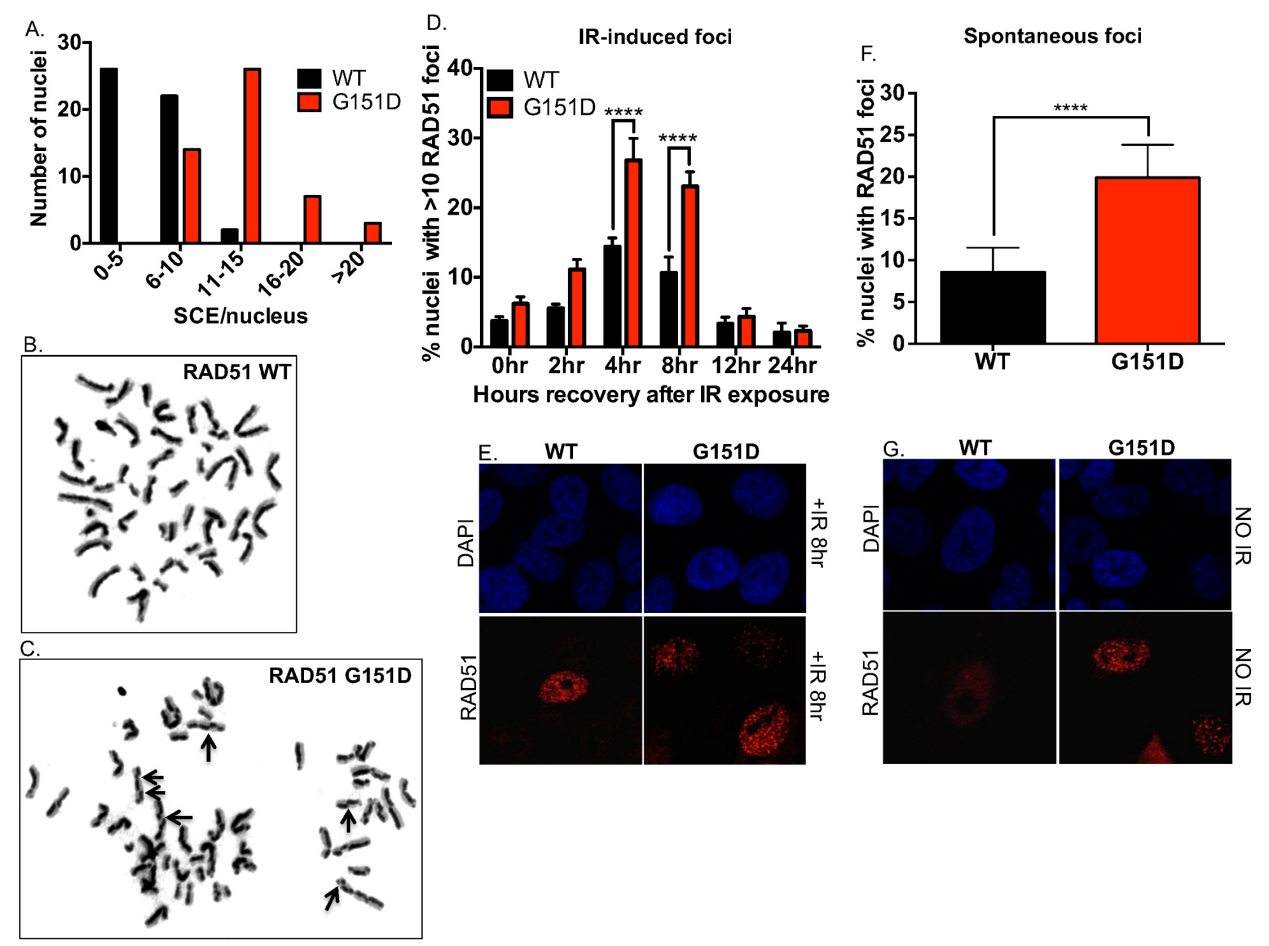
Increased number of spontaneous SCEs in MCF10A cells expressing RAD51 G151D as compared to WT expressing cells. **A.** Graphical representation of the distribution of the numbers of SCEs per nucleus. A total of 50 metaphases were analyzed from RAD51 WT and G151D expressing MCF10A pools. Graphed are the number of metaphases with 0–5, 6–10, 11–15, 16–20, and >20 SCEs for MCF10A RAD51 WT and G151D expressing pools. Representative images of nuclei of MCF10A cells expressing RAD51 WT **(B)** or RAD51 G151D **(C)**. **D,E.** MCF10A pools expressing RAD51 WT or G151D were exposed to 8GY ionizing radiation and immunofluorescence was performed at 0, 2, 4, 8, 12 and 24 hours post-IR exposure. **F,G.** MCF10A pools expressing RAD51 WT or G151D were labeled using a RAD51 antibody. Fluorescently labeled cells were visualized using a Zeiss LSM 510 META confocal imaging system and the number of nuclei with >10 RAD51 foci **(D,E)** (n> 500nuclei) or RAD51 foci **(F,G)** (n> 800 nuclei) was counted. **D,F.** The data are graphed as mean ± SEM. **** p< 0.0001. **E.** Representative images of RAD51 foci in MCF10A RAD51 WT and RAD51 G151D expressing pools at 8 hours post-IR exposure **G.** Representative images of RAD51 foci in MCF10A RAD51 WT and RAD51 G151D expressing pools.

As an outcome of HDR, elevated sister chromatid exchanges (SCEs) can be indicative of increased levels of HDR as well as increased illegitimate or inappropriately regulated HDR [38]. Therefore, we quantified the number of SCEs in MCF10A cells with stable and equivalent expression of RAD51 WT or G151D (Fig 1E). As shown in Fig 2A, MCF10A cells expressing RAD51 G151D exhibit increased levels of SCEs per nucleus as compared to WT expressing cells. To further emphasize the differences observed, cells expressing RAD51 G151D had 72% of nuclei with >10 SCEs in contrast to WT expressing cells with only 4% of nuclei with >10 SCEs. Representative images of stained metaphase spreads from RAD51 WT or G151D expressing cells are shown in Fig 2B and 2C, respectively. In combination with the HDR reporter results, our data suggest that cells expressing G151D have a hyper-recombinant phenotype.

RAD51 forms discrete nuclear foci in response to endogenous and exogenous DNA damage and these foci are considered sites of HDR repair of damage [39]. Previous studies have reported increased RAD51 foci in hyper-recombination models [40–43], therefore we investigated spontaneous and damage-induced RAD51 focus formation in RAD51 WT and G151D expressing MCF10A cells by immunofluorescence. MCF10A cells expressing G151D exhibited higher levels of damage-induced RAD51 foci at 4 and 8 hours post-IR exposure (Fig 2D and 2E), and spontaneous RAD51 foci (Fig 2F and 2G), as compared to WT-expressing cells. Additionally, there is an increase in the percentage of cells in S-phase in both damaged (after IR-exposure) and undamaged RAD51 G151D expressing cells as compared to WT expressing cells (S2 Fig). Accumulation of cells in S-phase and increased RAD51 foci in damaged and undamaged cells supports the hyper-recombination phenotype induced by RAD51 G151D.

### MCF10A cells expressing RAD51 G151D exhibit enhanced repair of IR-induced DSBs

**Fig 3:**
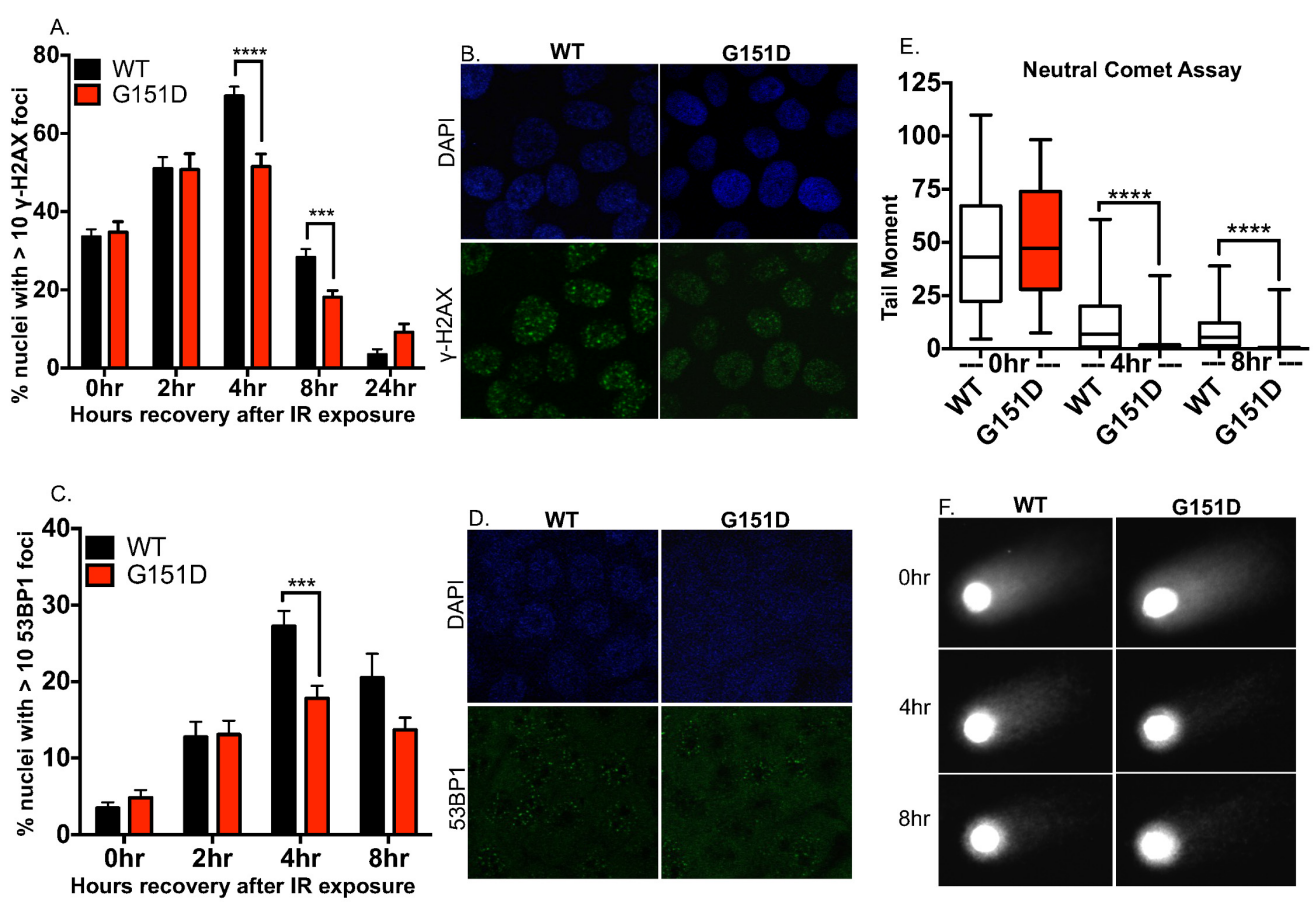
MCF10A cells expressing G151D repair IR-induced double strand breaks more rapidly than WT expressing cells. **A-D.** MCF10A pools expressing WT or G151D were exposed to 8GY ionizing radiation, fixed at 0, 2, 4, 8 and 24 (γH2AX only) hours post IR exposure and immunofluorescence was performed. Cells were labeled with a γH2AX antibody (green) **(A,B)** or 53BP1 antibody (green) **(C,D)**. Labeled cells were visualized using confocal microscopy. **A,C.** The number of nuclei with >10 foci of γH2AX or 53BP1 was counted. The data are graphed as mean ± SEM (n>500 nuclei) **** p< 0.0001; *** p<0.001. **B,D.** Representative images of γH2AX foci **(B)** or 53BP1 **(D)** at 4 hours post-IR exposure in MCF10A RAD51 WT or RAD51 G151D expressing pools. **E, F.** MCF10A pools expressing RAD51 WT or G151D were exposed to 8GY ionizing radiation then allowed to recover for 0, 4 and 8 h. Cells were harvested and single cell electrophoresis was performed to quantitate DNA damage using the comet assay. **E.** Data are graphed as mean ± SEM (number of nuclei counted per group: WT 0hr; 76, G151D 0hr; 80; WT 4hr; 72, G151D 4hr; 97, WT 8hr; 91, G151D 8hr; 100). **** p< 0.0001. **F.** Representative images from each time point of recovery post IR-exposure.

To visualize DSB repair after exposure to IR, we monitored gamma-H2AX (γ-H2AX) and p53 binding protein 1 (53BP1) foci using immunofluorescence. Phosphorylation of H2AX occurs rapidly when DSBs are present; the resultant γ-H2AX product forms foci at sites of DSBs, participating in the recruitment of DNA repair proteins to the break site. Similarly, 53BP1 rapidly forms discreet foci upon IR exposure at sites of DSBs, as compared to the diffuse localization demonstrated in undamaged cells. Therefore, both γ-H2AX and 53BP1 are surrogate markers for DSBs. MCF10A cells expressing RAD51 G151D had significantly fewer γ-H2AX foci compared to WT cells at 4 and 8 hours post IR exposure (Fig 3A and 3B) as well as significantly fewer 53BP1 foci at 4 hours post IR (Fig 3C and 3D). Although γ-H2AX and 53BP1 are well-established marker for DSBs, we also provide direct physical evidence for DSB repair by utilizing the neutral comet assay to compare the kinetic resolution of breaks by RAD51 WT and G151D expressing cells. The neutral comet assay utilizes single cell gel electrophoresis to detect DSBs by evaluating the DNA tail produced after voltage is applied to lysed cells embedded in agarose. MCF10A cells expressing RAD51 G151D had significantly shorter DNA “comet” tails at 4 and 8 hours post IR exposure as compared to WT expressing cells, indicative of fewer DSBs (Fig 3E and 3F). Notably, analysis of untreated cells with the neutral comet assay shows that neither WT nor G151D expressing cells have large numbers of DSBs (S3 Fig), even though elevated levels of spontaneous γH2AX foci are observed in G151D expressing cells. Thus, few DSBs are present in untreated WT or G151D expressing cells, but the γH2AX results indicate that perhaps some type of altered chromatin structure is present in G151D expressing cells. Collectively, these data indicate enhanced repair of IR-induced DSBs by RAD51 G151D expressing cells.

### Exogenous expression of RAD51 G151D confers resistance to DNA damaging agents

**Fig 4:**
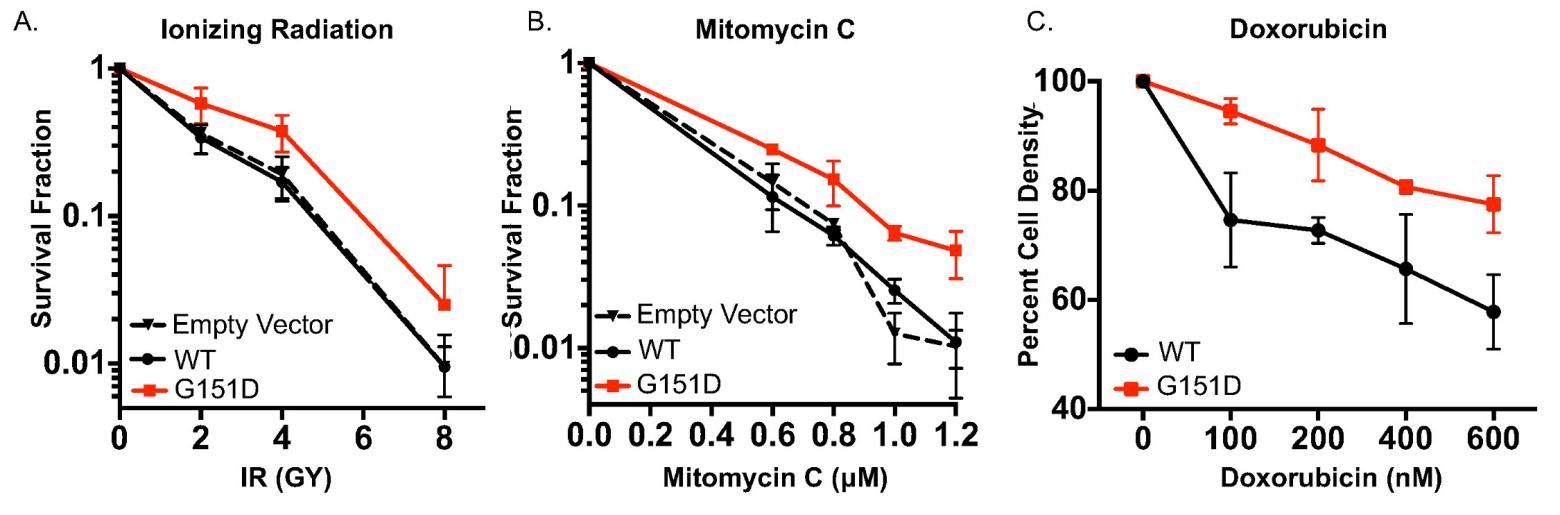
Expression of G151D confers enhanced resistance to DNA damaging agents. **A, B.** Serially diluted MCF10A empty vector, RAD51 WT and RAD51 G151D expressing pools were X-irradiated at 0, 2, 4, or 8 GY **(A)** or treated with 0 μM-1.2 μM Mitomycin C (MMC) **(B)**. After 10 days, colonies were stained with crystal violet and scored. Data are representative of 4 independent experiments and graphed as mean ± SD. **C.** MCF10A pools expressing RAD51 WT and G151D were plated in 48 well plates in triplicate and treated 24hrs later with 0 nM-600 nM doxorubicin for 1hr. Cell viability was measured 96hrs post-treatment by MTT assay. Data are representative of 4 independent experiments and graphed as mean ± SD.

**Fig 5:**
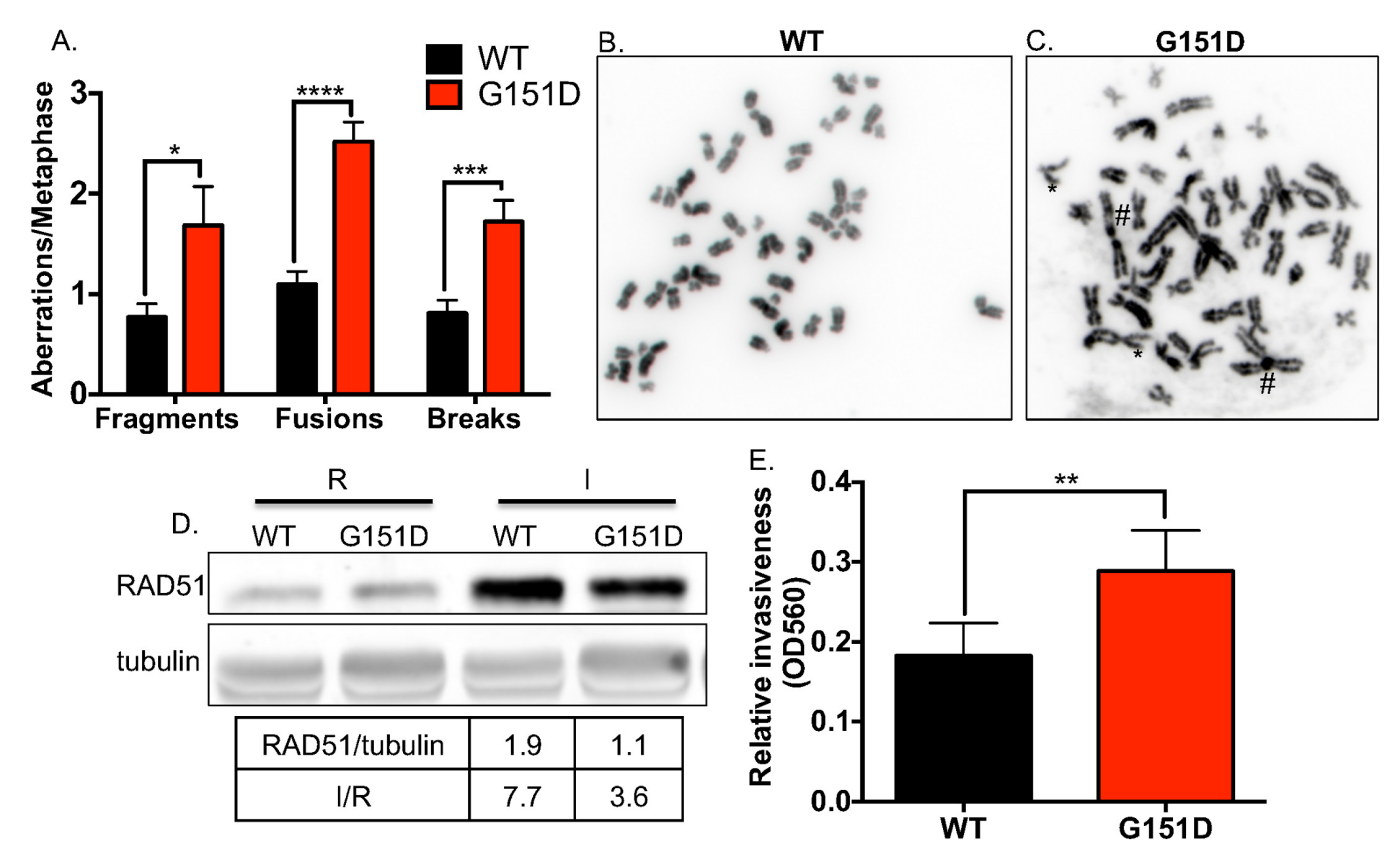
Increased genomic instability in MCF10A cells expressing RAD51 G151D. Metaphase spreads were prepared from undamaged, asynchronous RAD51 WT or G151D expressing MCF10A cells. **A.** Number of aberrations per metaphase. At least 50 metaphase spreads were scored for each cell line. ****p<0.0001, ***p<0.001, *p<0.05. **B.** Representative metaphase spread of MCF10A expressing RAD51 WT **(B)** or G151D **(C)**. **D.** Western blot demonstrates equivalent expression of exogenous RAD51 WT and G151D (I) in their respective MCF-7 pools, as well as the fold increase in expression over endogenous RAD51 expression (I/R). **E.** Cell invasion of MCF7 cells expressing either RAD51 WT or G151D was performed as described in Materials and Methods. Data are graphed as mean ± SD from 2 independent experiments. ** p<0.01.

Enhanced repair via HDR is likely to confer resistance to DNA damaging agents that result in DSBs. From a translational perspective, the tumor from which RAD51 G151D was identified turned out to be largely refractory to a range of therapeutic interventions including ionizing radiation (IR) and mitomycin C (MMC). Therefore, we characterized the response of MCF10A cells expressing either RAD51 WT or RAD51 G151D to IR, MMC, and doxorubicin. IR can lead to both DNA SSBs and DSBs through the formation of hydroxyl radicals or by direct ionization. MMC is an alkylating agent that forms interstrand DNA cross-links leading to inhibition of DNA replication and collapse of replication forks thereby resulting in the formation of DSBs [44]. Cells expressing RAD51 G151D exhibit increased clonogenic survival upon exposure to IR and MMC as compared to WT expressing cells (Fig 4A and 4B, respectively). There is no observable difference in sensitivity to IR or MMC between MCF10A empty vector expressing cells and WT expressing cells (Fig 4A and 4B, respectively). To rule out cell line specific effects, we generated MCF-7 cells with stable and equivalent expression of RAD51 WT or G151D (see Fig 5D for a western blot demonstrating expression levels of the exogenous proteins). Analogous to the increased resistance observed in MCF10A RAD51 G151D expressing cells, MCF-7 cells expressing RAD51 G151D exhibited increased resistance to IR as compared to RAD51 WT expressing cells in a clonogenic survival assay (S4 Fig). These results demonstrate that expression of RAD51 G151D in both non-transformed and transformed human cells results in enhanced resistance to damaging agents. Doxorubicin intercalates into DNA and inhibits topoisomerase II leading to the formation of both SSBs and DSBs. Clonogenic survival assays in MCF10A cells were difficult to interpret due to the highly cytotoxic and cytostatic nature of this compound, and therefore, the MTT [3-(4,5-dimethylthiazol-2-yl)-2,5-diphenyltetrazolium bromide] assay was employed to differentiate the response of G151D compared to WT cells. The MTT assay measures the conversion by metabolically active cells of water soluble MTT to formazan, an insoluble purple precipitate, and therefore assays for cell viability [45–47]. As shown in Fig 4C, cells expressing RAD51 G151D exhibited increased survival in response to the cytotoxic effects of doxorubicin as compared to WT expressing cells. Collectively, these data demonstrate that expression of RAD51 G151D in two independent cell lines, MCF10A and MCF-7, increases resistance to DNA damaging agents as compared to RAD51 WT expressing cells.

### RAD51 G151D increases chromosomal aberrations and cell invasion

The data thus far indicate that expression of RAD51 G151D resulted in increased HDR *in vivo*. However, it remained unclear whether the fidelity of HDR is affected by RAD51 G151D expression. To address this question, we prepared metaphase spreads from early passage MCF10A cells expressing RAD51 WT or G151D and scored chromosomal aberrations. As shown in Fig 5A, MCF10A cells expressing RAD51 G151D had significantly higher numbers of fragments, fusions (as indicated by # in Fig 5C) and breaks (as indicate by * in Fig 5C). Increased spontaneous chromosomal rearrangements in RAD51 G151D expressing cells suggests faulty, error-prone repair. Chromosomal rearrangements can contribute to genomic instability, potentially contributing to therapeutic resistance and tumor progression. The patient from whom this mutation was identified developed aggressive metastatic disease, therefore we postulated that RAD51 G151D may have contributed to the acquisition of the invasive and eventual metastatic behavior of the tumor cells. To address this hypothesis, we measured changes in cell invasion using a transwell system. MCF-7 cells expressing RAD51 WT or G151D (Fig 5D) were plated in a Boyden chamber with a bottom filter that is lined with matrigel, which mimics the extracellular matrix composition of the basement membrane. The chamber was then placed into a 6-well plate with FBS-containing media, acting as a chemo-attractant to stimulate chemotaxis. After 48 hours, cells that invaded through the matrigel were quantified. MCF-7 cells expressing RAD51 G151D exhibited significantly increased invasive potential as compared to MCF-7 cells expressing WT RAD51 (Fig 5E). Taken together, these data indicate expression of RAD51 G151D increased chromosomal aberrations as well as the invasive potential of MCF-7 cells. These results indicate that in addition to inducing a hyper-recombination phenotype, RAD51 G151D expression may be promoting illegitimate or error-prone HDR with mutational consequences.

### RAD51 G151D exhibits enhanced DNA strand exchange in the presence of RPA

**Fig 6:**
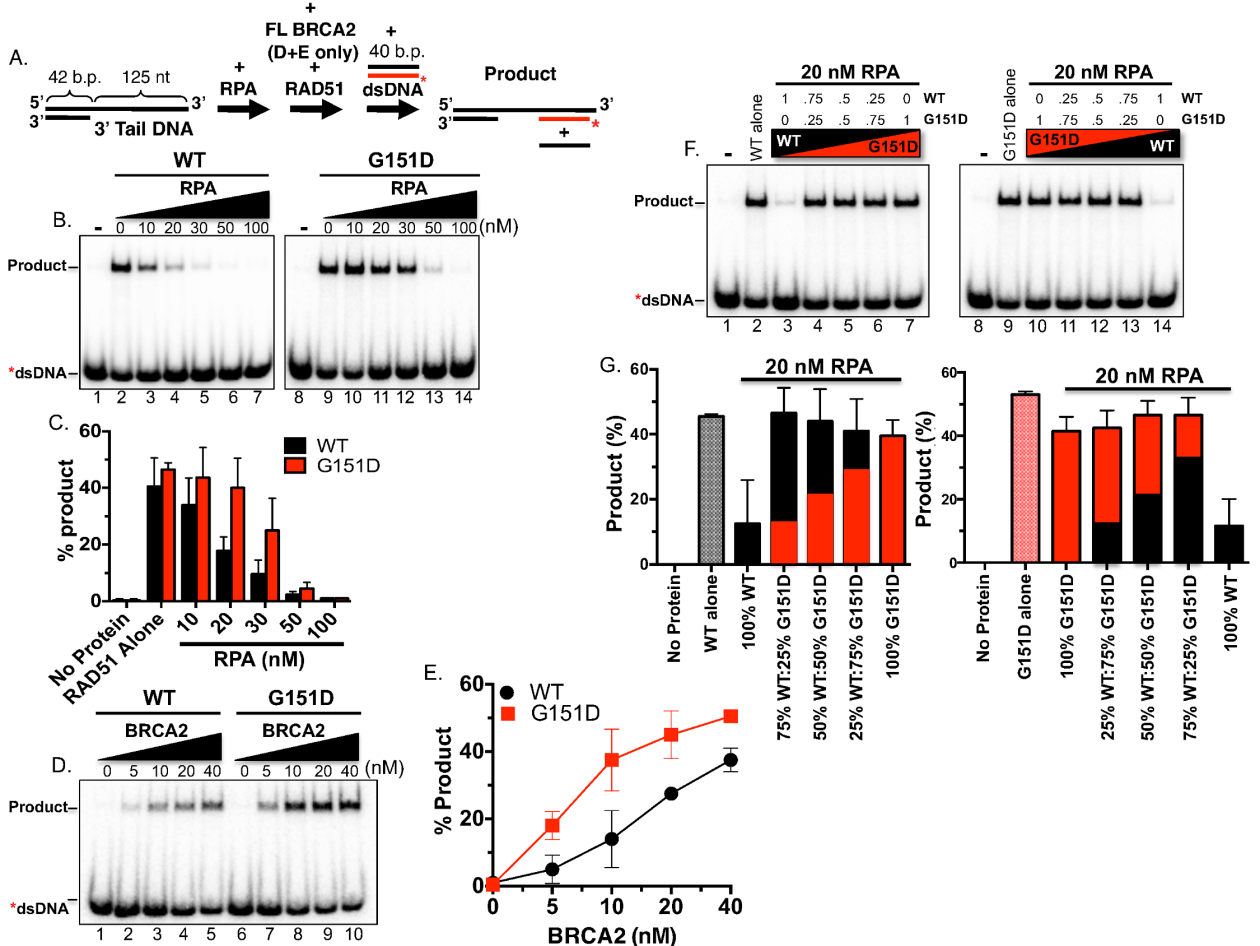
RAD51 G151D exhibits enhanced DNA strand exchange in the presence of RPA. **A.** Schematic of the DNA strand exchange assay. RPA was incubated first with the 3’ tail DNA followed by addition of RAD51 (BRCA2 and RAD51 were added simultaneously in D & E) and finally the radiolabeled donor DNA was added to initiate the reaction. **B.** Autoradiograms of DNA strand exchange assays comparing RAD51 WT to G151D in the presence of increasing concentrations of RPA. Lanes 1 and 8 are no protein controls. Lanes 2 and 9 contain either RAD51 WT or G151D in the absence of any other protein. **C.** Quantification of the PAGE gels shown in **(B). D.** Autoradiograms of DNA strand exchange assays performed in the presence of increasing concentrations of BRCA2 utilizing a fixed concentration of RPA (100nM) and of RAD51 WT or G151D (300 nM). **E.** Quantification of the gel shown in **(D)**. Mean values from three independent experiments were plotted. Error bars represent S.D. **F.** Autoradiograms of DNA strand exchange assays utilizing a 3’ tail DNA substrate in the absence or presence of pre-incubation with RPA. No protein controls (lanes 1 & 8). WT or G151D RAD51 in the absence of RPA (lanes 2 & 9). WT or G151D RAD51 added after pre-incubation of 3’ tail DNA with 20 nM RPA (lanes 3 & 10). WT and G151D mixed together at the depicted ratios and added after 20nM RPA (lanes 4–6 & 11–13). WT or G151D RAD51 added after RPA as in lanes 3 & 10 (lanes 7 & 14). The total RAD51 (WT+G151D) concentration in each reaction was kept constant. **G.** Quantification of autoradiograms in (A). Black bars represent WT protein and red bars represent G151D protein. Proportion of black bar:red bar in graphs represent of WT:G151D ratios.

The mechanism by which RAD51 G151D induces a hyper-recombination phenotype was pursued with particular interest since the mutation is not located in regions vital to RAD51 recombinase function, including the single- and double-stranded DNA binding surfaces (loops L1 and L2) or Walker A and B motifs. The location of the mutation in a loop on the outer surface of the RAD51 monomer and RAD51 filament indicated the potential for changes in protein-protein contact. We initially hypothesized that RAD51 G151D may possess altered affinity for pro-recombination mediators such as BRCA2 or PALB2 resulting in increased loading efficiency of RAD51 G151D onto ssDNA. However, as shown in S5 Fig, both RAD51 WT and G151D exhibited comparable binding affinities for BRCA2 and PALB2. Therefore, we decided to take a closer look at DNA strand exchange efficiency. DNA strand exchange measures the ability of RAD51 to nucleate on an ssDNA substrate and catalyze the subsequent invasion and pairing to a homologous duplex donor DNA. We employed an oligonucleotide strand exchange assay previously developed to monitor the mediator activity of BRCA2 (see schematic in Fig 6A) [48]. We purified both the WT and the mutant G151D RAD51 proteins and a direct comparison of the two by protein titration revealed comparable activities (see S6 Fig, WT and G151D protein titration in absence of RPA). RPA poses a blockade to RAD51-mediated DNA strand exchange when oligonucleotide substrates are utilized enabling the detection of mediator activity by proteins such as BRCA2. Surprisingly, by incubating a 3’ tailed DNA substrate with RPA first, the G151D protein promoted significant strand exchange activity under conditions that effectively block activity of the WT protein (compare lanes 3, 4 and 5 to 10, 11, and 12, respectively, in Fig 6B and 6C). In concordance with this result, co-incubation of G151D with BRCA2 resulted in a much higher level of activity compared to WT RAD51 (Fig 6D and 6E).

Enhanced DNA strand exchange in the presence of RPA provides a plausible mechanistic framework for the hyper-recombination phenotype observed in cells expressing the G151D variant. However, in our cell models, the G151D variant is expressed in the presence of endogenous RAD51 potentially mimicking the heterozygous state present within the patient’s tumor. We previously demonstrated that RAD51 WT and G151D directly interact in a yeast two-hybrid system and presumably assemble into mixed filaments in a biochemically reconstituted EMSA analysis [30]. Taken together, it is reasonable to assume that heterogeneous filaments, comprised of endogenous RAD51 and exogenous G151D, are capable of forming in cells expressing G151D. We measured DNA strand exchange activity using a mixture of WT and G151D purified proteins in various ratios using the same experimental protocol as described above. As previously shown, WT and G151D alone (in the absence of RPA) exhibited similar DNA strand exchange activities (Fig 6F, lanes 2 & 9), whereas G151D stimulated higher levels of strand exchange than WT in the presence of RPA (Fig 6F, compare lanes 3 & 10). Surprisingly, at all ratios tested, G151D mixed with WT increased DNA strand exchange to similar or slightly higher levels than G151D alone in the presence of RPA (Fig 6F and 6G). Even at 25% G151D to 75% WT, DNA strand exchange trended higher than G151D alone (Fig 6F lanes 4 & 13, Fig 6G). These data indicate that G151D incorporation into the RAD51 filament at equimolar or lower concentrations relative to WT improves the quality or stability of the overall filament leading to enhanced DNA strand exchange activity. Together, the mixed filament data demonstrated a range of G151D expression levels was sufficient to drive elevated DNA strand exchange activity indicative of the biochemical mechanism underlying the hyper-recombination phenotype observed in cells.

### RAD51 G151D forms altered filament species on ssDNA using smFRET analysis

**Fig 7:**
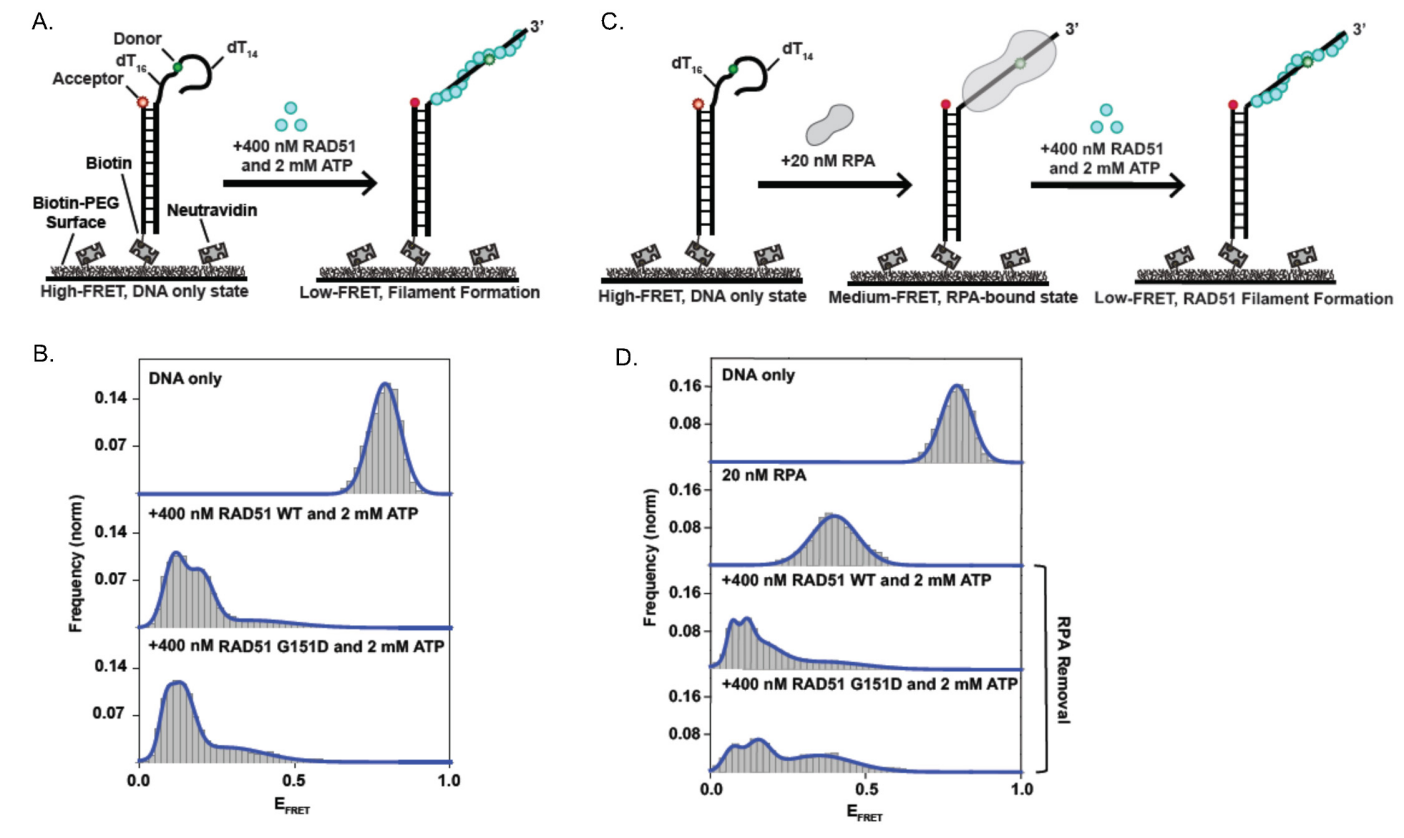
Single-molecule FRET assay for RAD51 WT- and RAD51 G151D- filament formation and RPA-RAD51 interaction. **A.** Illustration of single-molecule RAD51-ssDNA filament assay with partial DNA duplex containing a 30—nucleotide tail. Upon RAD51 filament formation, there is a transition from high FRET (DNA-only) to low FRET (RAD51-bound). **B.** Histograms display a clear shift to low FRET upon addition of 400 nM RAD51 and 2 mM ATP with increased stability using the RAD51G151D mutant. Histograms were generated after subtracting the zero FRET values and truncating photobleached portions of FRET trajectories. A minimum of 75 smFRET trajectories was used to generate each histogram. **C.** Illustration of RPA disruption assay where 20 nM RPA is bound to DNA and then 400 nM RAD51 + 2 mM ATP is added in an attempt to create RAD51 filaments. **D**. RPA-bound DNA leads to a distinct medium FRET state as opposed to the low FRET state observed upon RAD51 filament formation (Fig 7B, Panel 2). There is a transition to low FRET upon addition of 400 nM RAD51 (or RAD51 G151D) and 2 mM ATP (Panels 3 and 4), indicating that both RAD51 WT and RAD51 G151D successfully disrupt bound RPA-DNA interactions and assemble filaments. However, RAD51 WT led to a tighter peak, indicating more efficient RPA removal as compared to the RAD G151D mutant.

In order to investigate differences in filament flexibility or stiffness, we performed single molecule fluorescence resonance energy transfer (smFRET) (see [49] for a detailed smFRET methodology). Although we observed no difference in the CD spectra of RAD51 WT and G151D, which measures changes in folding of the RAD51 protein monomer [30], it is possible that the G151D mutation could propagate subtle structural changes throughout the filament, altering the physical properties of the filament. RAD51 WT and G151D filaments on ssDNA both exhibited a zigzag pattern that is characteristic of RAD51-DNA filaments, as seen by electron microscopy (EM) [30]. RAD51 G151D, however, qualitatively formed filaments with a more smooth coiled appearance as compared to RAD51 WT filaments indicating G151D filaments may have structural differences compared to WT filaments [30]. smFRET measures the relative fluorescence intensity of a donor fluorophore (green), placed in the ssDNA and an acceptor fluorophore (red), present in the duplex DNA. When the donor and acceptor fluorophore are within close proximity, the donor fluorophore transfers its energy to the acceptor fluorophore, and therefore, only the acceptor fluorophore is detected (red channel); this is termed a high FRET state with a FRET efficiency (E_FRET_) approaching 1.0 (Fig 7A). When the ssDNA is maximally extended, the distance between the donor and acceptor fluorophores is increased, and therefore, only the donor fluorophore will be detected (green channel), resulting in a low FRET state with an E_FRET_ approaching 0 (Fig 7A and 7B). The relative fluorescence of the donor and acceptor fluorophore is directly proportional to the distance between them. Therefore, changes in RAD51 filament properties on the ssDNA tail will be reflected in changes in the distance between the donor and acceptor fluorophores (measured by changes in the relative fluorescence, and corresponding E_FRET_). The addition of 400 nM of RAD51 WT or G151D in the presence of 2 nM ATP decreases the E_FRET_, indicating efficient filament formation on the ssDNA tail (Fig 7B). However, there are clearly 2 peaks in the RAD51 WT and a broader E_FRET_ distribution (Fig 7B). In contrast, RAD51 G151D exhibits only 1 peak with a very narrow E_FRET_ distribution (Fig 7B). These data suggest that RAD51 G151D forms a different, more stable, filament species than RAD51 WT.

**Fig 8:**
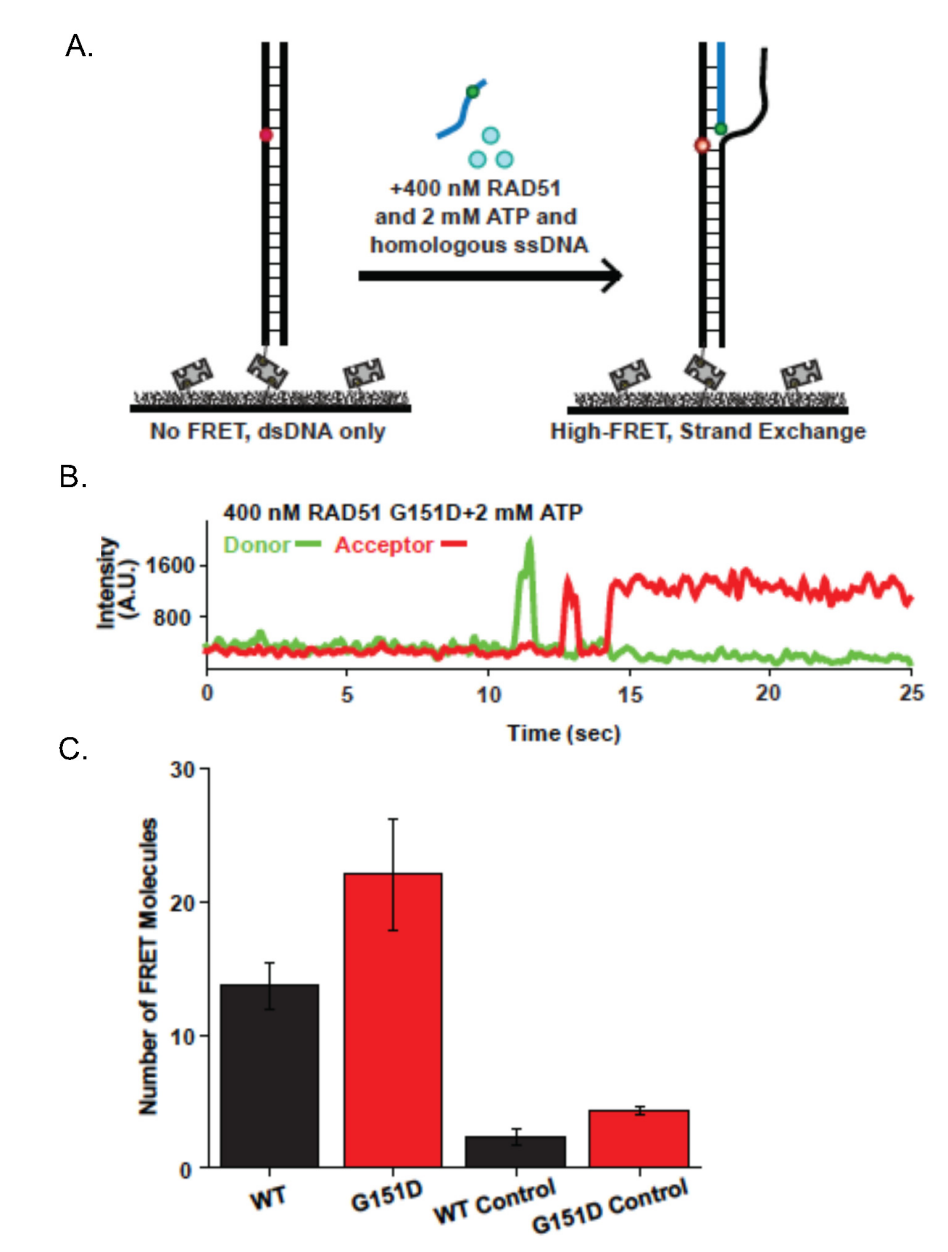
Single-molecule visualization of RAD51 strand exchange. **A.** Illustration of single-molecule strand exchange assay. FRET is not observed until homologous single-stranded DNA (ssDNA) invades duplex DNA tethered to the surface. **B.** Representative smFRET trajectory for strand exchange assay in the presence of 400 nM RAD51 G151D, 2 mM ATP, and 1 nM homologous ssDNA. Increase in acceptor intensity (red) indicates homology detection and strand exchange. **C.** RAD51 G151D has increased strand exchange efficiency as compared to wild-type. Strand exchange efficiency was measured as the average number of smFRET pairs per imaged area (error bars = SEM.; n>3). As a control, strand exchange experiments were run with non-homologous ssDNA substrates. For all reactions, protein was incubated for 60 minutes.

To determine the efficiency of RPA displacement of RAD51 G151D as compared to RAD51 WT, we pre-loaded the ssDNA tail with 20 nM RPA and performed smFRET analysis with 400 nM RAD51 WT or G151D and 2 nM ATP (Fig 7C). As shown in Fig 7D, RPA binding alone to the ssDNA tail results in an intermediate FRET state. Surprisingly, RAD51 WT more effectively displaced RPA from the ssDNA tail as seen by the predominant shift of the intermediate E_FRET_ peak when RPA is bound to a low E_FRET_ peak upon RAD51 WT filament formation (Fig 7D). In contrast, a prominent intermediate E_FRET_ peak persisted after the addition of RAD51 G151D to the RPA-bound smFRET substrate, indicating that a higher proportion of RPA remained bound to the ssDNA (Fig 7D). These results suggest that the enhanced DNA strand exchange activity cannot be attributed to increased efficiency in RPA displacement but perhaps RAD51 G151D may be better at promoting the strand invasion and pairing reaction than WT. To test this hypothesis, we measured the strand invasion and pairing reaction by smFRET, using a donor labeled ssDNA substrate that has sequence homology with a short section of surface tethered duplex DNA substrate containing the acceptor fluorophore (Fig 8A). The acceptor fluorophore is positioned in the duplex DNA such that upon strand invasion and pairing between the homologous sequences by the RAD51-bound ssDNA, the donor and acceptor fluorophores are within close proximity resulting in fluorescence detectable by the acceptor fluorophore only (red channel) (Fig 8A; representative smFRET trajectory shown in Fig 8B). As shown in Fig 8C, RAD51 G151D exhibited increased strand exchange efficiency as compared to RAD51 WT. As a control, strand exchange was measured using a non-homologous ssDNA substrate containing the donor fluorophore. Interestingly, RAD51 G151D also exhibited increased strand exchange activity with the non-homologous ssDNA substrate compared to RAD51 WT (Fig 8C), providing additional evidence that RAD51 G151D may promote error-prone or illegitimate HDR (as suggested by data in Figs 2 and 5). Collectively, these data indicate that RAD51 G151D possesses significantly higher DNA strand exchange efficiency compared to RAD51 WT.

### RAD51 G151D expression increases replication fiber tract length

**Fig 9:**
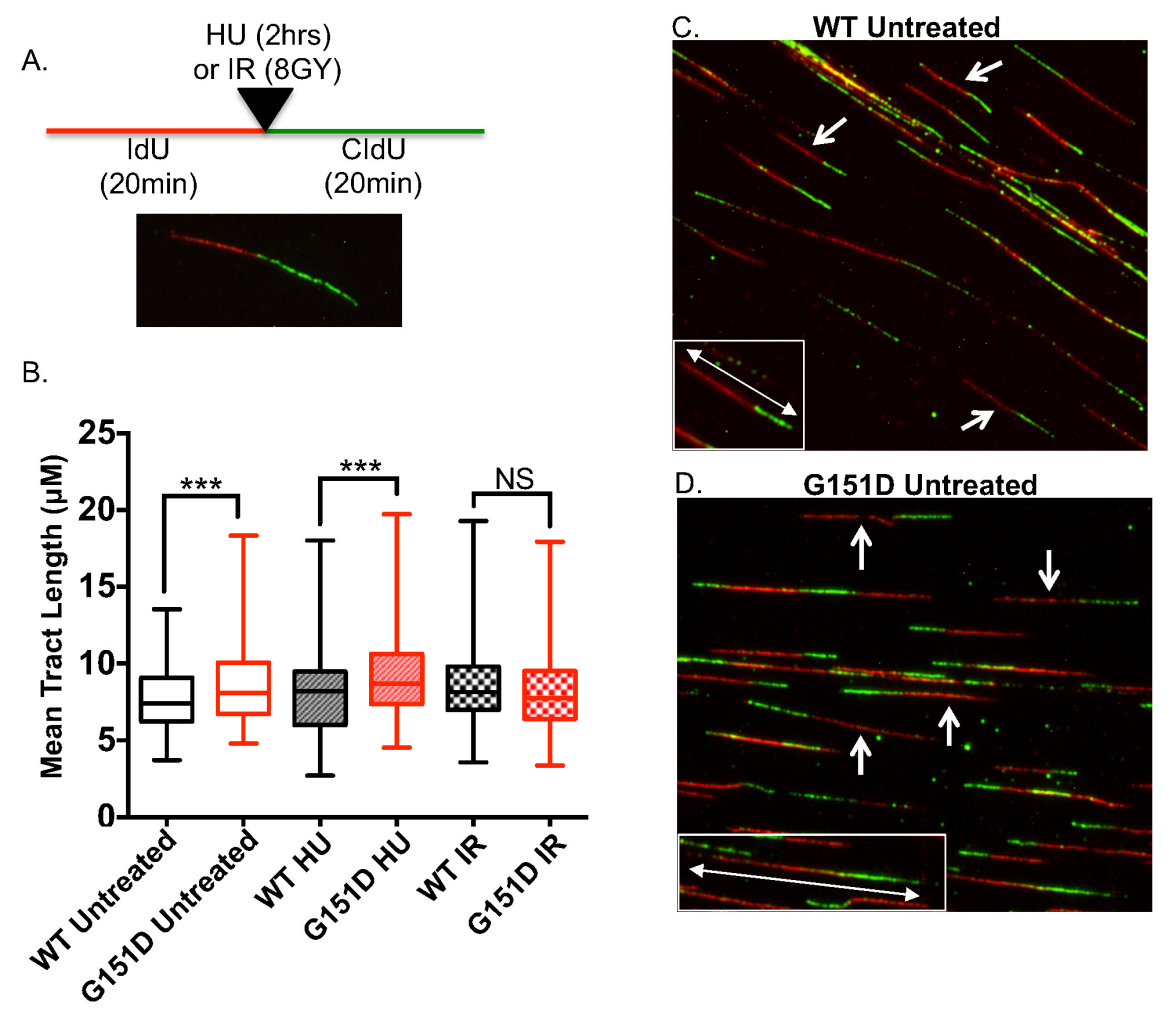
Increased replication fork tract length in MCF10A cells expressing RAD51 G151D. **A.** Schematic of the experimental setup for the DNA fiber assay and a representative image of an elongating replication fork, which was the replication structure exclusively used to measure replication tract length. **B.** Replication tract length of elongating replication forks measured by DNA fiber assay in untreated, HU-treated (0.5mM for 2 hrs), or IR-treated (8GY) MCF10A cells expressing RAD51 WT or G151D. >100 replication tracts were measured for MCF10A RAD51 WT or G151D expressing cells for each treatment group. Data are representative of 3 independent experiments. Graphed as mean ± SD. **C,D.** Representative images of images of untreated MCF10A RAD51 WT or G151D expressing cells. Arrows indicate representative elongating replication fork structures used to measure replication tract length. Insets highlight representative replication tracts for RAD51 WT **(C)** and RAD51 G151D **(D)** cells.

The hyper-recombinant activity of RAD51 G151D, observed both *in vivo* and *in vitro* herein, would suggest that RAD51 G151D expression might have an effect on replication fork maintenance. Therefore, we performed the DNA fiber assay and measured replication tract length. MCF10A cells expressing RAD51 WT or G151D were pulsed with iododeoxyuridine (IdU) for 20 min followed by treatment with 0.5 mM hydroxyurea (HU), IR (8 GY) or untreated then pulsed with chlorodeoxyuridine (CIdU) for 20 min (Fig 9A). IdU and CIdU are analogs of thymidine and therefore can get incorporated into newly synthesized DNA. Incorporated IdU or CIdU into newly synthesized DNA can be detected using IdU- or CIdU-specific primary antibodies, producing a red tract (IdU) followed by a green tract (CIdU) (Fig 9A). As shown in Fig 9B, RAD51 G151D expression significantly increases replication tract length in untreated and in HU treated cells as compared to cells expressing RAD51 WT. However, upon induction of DSBs by IR, there is no significant difference in tract lengths of G151D-expressing cells compared to WT expressing cells (Fig 9B). These data indicate that RAD51 G151D may bind more extensively to ssDNA present during replication, thereby increasing replication tract length.

## Discussion

Here we show that the G151D tumor-associated RAD51 variant induces a hyper-recombination phenotype. We demonstrate that expression of RAD51 G151D leads to increased levels of HDR in the DR-GFP assay, as well as the HDR luciferase reporter assay, and a high frequency of SCEs. In biochemical experiments we show that purified RAD51 G151D protein catalyzes higher levels of DNA strand exchange activity than the WT protein in the presence of RPA. Our smFRET analysis suggests that G151D forms a discrete species indicative of a stable filament and is more efficient at the strand invasion and homology search steps than the WT protein. In addition, DNA fiber assays show that expression of RAD51 G151D leads to increased tract length of replication fibers. RAD51 G151D was identified as a heterozygous somatic breast cancer variant and the patient harboring the G151D RAD51 variant appeared to be resistant over the course of three years to various chemotherapeutic drugs including doxorubicin (Adriamycin), mitomycin C, and 5-fluorouracil, along with ionizing radiation (IR), eventually succumbing to metastatic disease. In light of the hyper-recombination phenotype induced by RAD51 G151D presented in this study, we suggest that RAD51 G151D contributed to the refractory and aggressive nature of the breast cancer from which it was identified.

### Hyper-recombination by G151D

The DNA strand exchange activity of RAD51 G151D was higher than WT in both the oligonucleotide DNA strand exchange assay and the smFRET analysis, providing a mechanistic explanation for the hyper-recombination phenotype observed in cellular assays, including increased RAD51 foci, increased HDR, and enhanced DSB repair. Gain-of-function mutations in yeast Rad51, predominantly in the L2 loop in Rad51, were previously shown to decrease sensitivity to IR in *rad55* and *rad57* mutant yeast strains [50]. The Rad51 paralogs, Rad55 and Rad57, are recombination mediators that have been shown to stimulate DNA strand exchange by promoting Rad51 nucleation onto RPA-bound ssDNA [51]. Similar to the enhanced DNA strand exchange activity exhibited by RAD51 G151D, the Rad51 I345T gain-of-function mutant also increased DNA strand exchange activity [50,52]. Whereas the hyper-recombinant activity of Rad51 I345T was attributed to increased binding affinity for single- and double-stranded DNA, we detected no difference in DNA binding affinities between RAD51 G151D and RAD51 WT (S7 Fig) [30]. Increased pairing and strand exchange reaction was observed using both the oligonucleotide DNA strand exchange activity assay and smFRET analysis, which utilize short ssDNA substrates (<126bp). In contrast, there was no difference in strand exchange activity between RAD51 WT and G151D using the ϕX174 virion DNA which measures strand exchange activity using a >5kb ssDNA as a substrate [30]. Furthermore, G151D expression increased replication tract length in untreated and HU-treated cells, suggesting that RAD51 G151D may be stabilizing elongating replication forks by binding to ssDNA at the fork. In combination our data suggest that G151D may not require the extensive strand resection or ssDNA sequence needed by WT RAD51 to associate with DNA and engage in strand exchange activities.

Increased filament stability is another possible contributing factor to the hyper-recombinant activity of RAD51 G151D. We previously demonstrated a 6-fold decrease in catalytic efficiency of ATP hydrolysis by RAD51 G151D compared to WT [30]. The decreased catalytic turnover of the RAD51 G151D variant may provide a more stable ATP-bound active filament. The smFRET analysis of filament flexibility and stiffness may provide experimental evidence of a more stable filament formed by RAD51 G151D. Clearly, WT and G151D form different filament species on the ssDNA tail, as seen by the different peak shapes and E_FRET_ distributions produced by the respective proteins. In conjunction with the measured decrease in ATP hydrolysis, it is conceivable that G151D may form a more stable filament. A more stable RAD51 filament could promote the formation of double Holliday junctions (dHJs) resulting in increased crossover events (increased SCEs) with mutagenic outcomes (increased chromosomal aberrations).

### Biological consequences of a hyper-recombinant RAD51 protein

HDR is a cell cycle regulated process utilizing multiple mechanisms at each step to prevent inappropriate engagement or erroneous recruitment of HDR proteins to the site of DNA damage [53,54]. Inappropriate engagement of HDR during G_1_, utilization of imprecise templates (e.g. homologous chromosome or repetitive sequences), and improper dissolution of Holliday junction intermediates can lead to mutations, loss-of-heterozygosity, and other genomic abnormalities [35,55–57]. For instance, disruption of the Bloom (BLM) and Werner (WRN) genes, RecQ helicases that function to suppress inappropriate recombination, leads to increased chromosomal rearrangements and most notably, increased sister chromatid exchanges (SCEs) [3,40,58–60]. In a similar manner, we demonstrate that expression of RAD51 G151D in human cells generates chromosomal aberrations and SCEs. Given the parallel phenotypes, as well as the increased strand exchange activity using a non-homologous substrate in the smFRET analyses, perhaps RAD51 G151D initiates HDR at inappropriate times during the cell cycle or facilitates strand invasion into inappropriate substrates. Glycine 151 is located on the outer surface of both the RAD51 monomer as well as the filament; therefore the G151D mutation may alter the surface properties of RAD51-DNA filaments [30]. Changes at the surface of the RAD51 monomer and filament could alter interactions with proteins that regulate and/or enhance HDR. RAD51 G151D mediated DNA strand exchange was further enhanced by the presence of BRCA2, therefore it is possible that altered interactions with other recombination mediators could affect subsequent strand invasion, homology search stringency, or eventual displacement of RAD51 to facilitate DNA polymerase extension of the 3’ invading strand.

RAD51 G151D contributes significantly to increased genomic instability in both non-transformed and transformed cells. In this report we demonstrate increased SCEs in cells expressing RAD51 G151D. Human mitotic cells preferentially process HDR intermediates in favor of non-crossover (NCO) events to prevent removal of genetic information in the form of loss-of-heterozygosity (LOH) [61–63]. Therefore, spontaneous SCEs are indicative of increased crossover (CO) events as a consequence of de-regulated HDR. It has previously been shown in yeast that lack Sgs1 (the yeast BLM ortholog), a vital component of the BTR complex that processes dHJs via dissolution giving rise to NCO recombinants only, have increased SCEs [62,64]. In the absence of proteins necessary for branch migration and dissolution of dHJs, cells can utilize an alternative HJ processing mechanism giving rise to both NCO and CO recombinants, increasing the levels of SCEs. Based on its genetic and biochemical attributes, perhaps G151D assembles filaments during the post-synaptic phase of SDSA resulting in re-formation of the D-loop and biased repair towards DSBR resulting in increased CO events.

### RAD51 G151D confers drug resistance

We propose that RAD51 G151D contributes significantly to the refractory phenotype of cancer cells, by conferring resistance to therapeutics as a result of its hyper-recombinant phenotype. We have shown that expression of RAD51 G151D in two independent human cell models confers increased resistance to DNA damaging agents. The patient from whom the *G151D* mutation was identified failed to respond to a number of therapeutics, including IR, MMC and doxorubicin. In total, our data lead us to conclude that RAD51 G151D expression directly contributed to the therapeutic resistance of the primary and metastatic tumors in the patient. Previous work has demonstrated multiple mechanisms by which tumor cells can acquire multidrug resistance [5,65–69]. Overexpression of RAD51 has been associated with radio- and chemo-resistance [33,34,70,71]. However, in this study, we provide the first evidence that RAD51-induced hyper-recombination is a mechanism of drug resistance in both normal mammary epithelial cells and a breast cancer cell line. Importantly, based on the mixing experiments of WT with G151D, and the resultant change in DNA strand exchange activity, we predict that low levels of G151D expression in a tumor would result in a hyper-recombination phenotype and the potential for therapeutic resistance. These findings have important clinical significance should the G151D variant be used as a guide for therapeutic intervention in the future.

Genetic diversity and adaptation in cancer cells is driven, in part, by loss-of-heterozygosity and copy number variation, which generate populations of cells with increased proliferative capacity, resistance to DNA damaging agents, and invasive and metastatic properties. We propose that RAD51 G151D increases the levels of cellular genomic instability and resistance to mainline DNA damaging agent therapy, driving the clinically aggressive disease exhibited by this patient. Utilization of the *RAD51 G151D* mutation as clinical guide for predicting therapeutic response and disease progression could have a significant impact on the survival outcome of cancer patients harboring this somatic tumor variant. Furthermore, our results provide support for future studies to identify RAD51 mutations and other HDR-associated variants associated with disease progression and therapy resistance. We anticipate that further detailed mechanistic insights into the role of HDR factors in cancer will lead to novel pharmaceutical targets and improved clinical outcomes for those patients with refractory tumors and metastatic disease.

## Materials and Methods

### Plasmids and cloning

The RAD51 G151D variant was generated as previously described [6]. The RAD51 WT and RAD51 G151D sequences were then amplified by PCR and cloned into the *NotI*, *BamHI* sites in the pRVY-Tet retroviral vector. The pCBA*IsceI* expression vector was a kind gift from the Jasin laboratory.

### Cell lines and cell culture

MCF10A cells, an immortalized, non-transformed mammary epithelial cell line, were obtained from ATCC. MCF10A cells were maintained in DMEM/F12 medium (Corning, Cellgro) supplemented with 5% horse serum (HyClone), 1% penicillin-streptomycin (Gibco), epidermal growth factor (EGF) (20 ng/mL, Peprotech), hydrocortisone (0.5 μg/mL, Sigma-Aldrich), insulin (10 μg/mL, Invitrogen), Cholera Toxin (100 ng/mL, Sigma-Aldrich). GP2-293 cells (Clonetech), a retroviral packaging cell line, MCF-7 DRGFP cells (a kind gift from the Jasin lab), and U2OS-DRGFP cells (a kind gift from the Bindra lab) were maintained in DMEM (Corning, Cellgro) 10% fetal bovine serum (FBS) (Invitrogen) supplemented with 1% penicillin-streptomycin. All cells were maintained at 37°C in a humidified 5% CO_2_ incubator. Pools of MCF10A, MCF-7, MCF-7 DRGFP, and U2OS-DRGFP cells with stable expression of RAD51 WT or RAD51 G151D were generated as previously described [72], using a TET-OFF inducible expression vector. All cells were maintained in 2 ug/mL doxycyline during selection with 200 ug/mL hygromycin. Once selection was completed, doxycycline was removed from the media to induce expression and cells were maintained in 15 ug/mL hygromycin. All experiments were performed using cells at or under passage 5 (post-removal of doxycycline from the media).

### Western blotting

Synchronized cells were collected in cell lysis buffer (150 mM NaCl, 50 mM Tris pH 7.8, 1% NP-40, 0.25% sodium deoxycholate) then centrifuged at 10,000xg for 10 minutes. Samples were mixed with 6x loading buffer (375 mM Tris-HCl, 9% SDS, 50% glycerol, bromophenol blue), separated on a 10% SDS-PAGE gel and transferred to polyvinylidene difluoride (PVDF-FL) membranes (Millipore, Billerica, MA, USA). The membrane was blocked in Odyssey blocking buffer (PBS) (Millipore) for 1hr at room temperature (RT) with gentle shaking. The membrane was then probed rabbit polyclonal RAD51 primary antibody (1/300) (Santa Cruz Biotechnology, H-92) and mouse monoclonal tubulin primary antibody (1/10000) (Abcam, DM1A) overnight at 4°C with gentle shaking. After 3 washes in PBS/0.1% Tween, the membrane was probed with IRDye 800CW goat anti-mouse IgG (H+L) (1/20,000) and IRDye 680RD goat anti-rabbit IgG (H+L) (1/20,000) for 1hr at RT. The membrane was washed with PBS/0.1% Tween and visualized using the Odyssey CLx Infrared Imaging System. Expression levels were quantified using Image Studio Software version 2.1.10.

### DR-GFP assay

MCF-7 DR-GFP cells expressing RAD51 WT or RAD51 G151D were plated at 2x10^6^ cells per well in a 6-well plate. The next day, the cells were nucleofected using the Amaxa Cell Line Nucleofector Kit V (Lonza) as per the manufacturers instructions with either no DNA, 2 μg of pmaxGFP, or 1–4 μg of the I-*Sce*I expression vector pCBA*Sce* [73]. The percentage of GFP-positive cells was quantified by flow cytometry 72 hours post-nucleofection on a Becton Dickinson FACSAria LSRII analytical cytometer and using FlowJo x software version 10.0.7v2. Genomic DNA was isolated 5 days after nucleofection in order to determine the percentage of I-*Sce*I site loss for cells nucleofected with no DNA or the I-*Sce*I expression vector. To amplify the DR-GFP sequence surround the I-*Sce*I site, PCR reactions with a final volume of 50 μL included: 1 μg of genomic DNA as a template, dNTPs (1 mM), MgSO_4_ (1 mM), Pfx amplification buffer (1x), PCRx Enhancer solution (1x), Platinum *Pfx* DNA Polymerase (Invitrogen) and primers at 0.2 μM. The primer sequences were DR-GFP-RS-F 5’ CGTGCTGGTTATTGTGCTGTCTCA and DR-GFP-RS-R 5’ TGCTGCTTCATGTGGTCGGGGTAG. Amplification was performed as previously described [36] using a BioRad C1000 Thermal Cycler. Following amplification, the PCR products were purified using a Qiagen PCR purification kit as per the manufacturers instructions. The purified PCR products were digested with 10 units of I-*Sce*I (NEB) for 20 hours, separated on a 2.5% agarose gel containing SYBR Safe DNA gel stain (Invitrogen) and visualized using the BioRad VersaDoc Imaging System. Signals of the +I-*Sce*I and–I-*Sce*I bands were quantified using Quantity One software version 4.6.5. The percent HDR was calculated by dividing the percent GFP+ cells by percent I-*Sce*I site loss.

### HDR luciferase assay

The HDR luciferase assay was performed as previously described [37]. Briefly, a reporter gene (gWiz.Lux-5’-3’Luc) was constructed from the parental vector gWiz Luciferase (Genlantis). An I-*Sce*I site was created in the luciferase ORF that completely disrupts luciferase activity. The second luciferase ORF inserted downstream lacks a promoter but can be utilized as a donor in HDR of the first luciferase ORF upon generation of a DSB by expression of the I-*Sce*I nuclease. The p*Sce*-MJ mammalian I-*Sce*I expression vector was a kind gift from Dr. Fen Xia. To perform the assay, cells were seeded into 6-well plates at 5x10^5^ cells/well. 24 hours later, cells were nucleofected with 500 ng of gWiz.Lux-5’-3’Luc vector and 500 ng of the I-*Sce*I expression vector using Amaxa Cell Line Nucleofector Kit L (program T020). As negative and positive controls respectively, gWiz.Lux-5’-3’Luc or gWiz. Lux vector were transfected alone. Cells were harvested 24 and 48 hours post-nucleofection. Luminescence was measured using an integration time of 5 seconds with 40 μL of the lysate plus 100 μL of luciferin substrate (One-Glo luciferase assay, Promega). Luciferase values were measured as independent triplicates in each experiment. The data presented is the average of two independent experiments.

### Sister chromatid exchange assay

The sister chromatid exchange assay was performed as previously described [74,75] with minor changes. Briefly, 24 hours after cells were plated, 20 μM bromodeoxyuridine (BrDu) (Sigma) was added to the plates for 72 hours. After 0.1 μg/mL of Colcemid (Invitrogen) was added to the plates for 3 hours, the cells were harvested by trypsinization and metaphase spreads were prepared as previously described [76]. The subsequent slides were dried for 3 days, rehydrated in 1xPBS then incubated with 25 μg/mL Hoechst 33258 (Sigma) for 20 minutes. Slides were mounted in equal volumes of 0.1 M Na_2_HPO_4_ and 0.1 M KH_2_PO_4_ (pH 6.8), sealed with rubber cement then placed under one 100-W lamp (Reveal; 100 W; 1,352 Lumens; A-19 Shape; General Electric) that was placed at a distance of 20 cm from the surface of the slides for 25 minutes. Slides were incubated in pre-warmed 1X SSC (20X SSC: 3 M NaCl, 300 mM sodium citrate) for 1hr at 50°C, washed in water then stained in 5% KaryoMax Giemsa (6.0 g Azur II Eosin and 1.6 g Azur II per liter in glycerol/methanol in equal volumes of 0.004 M Na_2_HPO_4_ and 0.004 M KH_2_PO_4_ (pH 6.8) (Invitrogen) for 20 minutes. Slides were washed in water, dried and mounted in Permount mounting media. Spreads were imaged under a 100x objective using an Olympus BX50 Light Microscope with QImaging Retiga 2000R digital camera and software.

### Chromosomal aberrations

Metaphase spreads were prepared and chromosomal aberrations were analyzed as described [76].

### Clonogenic survival assays

For ionizing radiation (IR) sensitivity and mitomycin C (MMC), cells seeded at various low densities were exposed to 0, 2, 4, 6, 8 GY of IR (X-irradiation) or 0, 0.6, 0.8, 1.0 or 1.2 uM of MMC for 4 hours then rinsed twice with PBS. The media was replaced with DMEM/F12 complete growth media and the cells were incubated for 10–12 days before being washed with 1xPBS and stained with crystal violet (0.5% crystal violet in 80% methanol). Colonies with more than 50 cells were scored by eye. For both IR and MMC experiments, plating efficiency was calculated by dividing the number of colonies counted by the number of cells plated; clonogenic survival was determined by dividing the plating efficiency of treated cells by the plating efficiency of untreated cells. The data is representative of 4 independent experiments.

### MTT assay

To measure doxorubicin sensitivity, 10^3^ cells were plated per well in triplicate in a 96 well plate. The following day, the cells were treated with 0, 100, 200, 400 or 600 nM for 1 hour after which the media was replaced with DMEM/F12 complete media. Cell viability was measured 72 hours post-treatment using the Vibrant MTT Cell proliferation assay kit (Invitrogen) as per the manufacturers instructions. Absorbance was measured using a BIO-TEK Synergy HT micro-titer plate reader. Percent cell death was calculated by dividing the absorbance of treated cells by the absorbance of untreated cells, subtracting that value from 1 then multiplying by 100. The data is representative of 4 independent experiments.

### Immunofluorescence assay

MCF10A RAD51 WT or G151D expressing cells plated in 8 well chamber slides (Millipore) were exposed to 0 or 8GY of IR (X-rays) then allowed to recover for 0, 2, 4, 8 or 24 hours post-exposure. Cells were washed 2 times with PBS then fixed (4% paraformaldehyde, 0.02% TritonX-100) for 15 minutes at room temperature. Cells were rinsed with PBS then incubated with blocking/permeabilization solution (10% normal goat serum, 0.5% TritonX-100) for 1 hour at with gentle shaking. The blocking/permeabilization solution was then replaced with blocking/permeabilization solution containing diluted primary antibody as follows; rabbit polyclonal RAD51: 1/100 (Santa Cruz, H-92) and mouse monoclonal anti-phospho-Histone H2A.X (Ser139): 1/200 (Millipore, clone JBW301) and incubated overnight at 4°C with gentle shaking. The next day, the cells were washed with PBS/0.5% TritonX-100 followed by 2 washes with PBS. Cells were then incubated with AlexaFlour 488 goat anti-mouse IgG (H+L) antibody (1/1000) (Invitrogen) and AlexaFlour 647 goat anti-rabbit IgG (H+L) antibody (1/1000) (Invitrogen) diluted in blocking/permeabilization solution for 1 hour with gentle shaking. The cells were washed with PBS/0.5% TritonX-100 followed by 2 washes with PBS then slides were mounted in Prolong Gold Antifade reagent with DAPI (Invitrogen). Cells were imaged using a Zeiss LSM 510 META confocal scanning laser microscope. For spontaneous foci formation, 1243 nuclei of MCF10A RAD51 WT expressing cells and 1168 nuclei of MCF10A RAD51 G151D expressing cells were counted. For RAD51 and gamma H2A.X foci formation post-IR exposure, at least 1500 nuclei were counted for both cell lines.

### Neutral comet assay

The Trevigen CometAssay was performed in order to measure double strand DNA breaks after exposure to ionizing radiation as per the manufacturers instructions. Briefly, 5 x 10^5^ cells were plated in 60 mm tissue culture dishes. The next day, the cells were exposed to 8GY of ionizing radiation (X-rays) or untreated and harvested by scraping at 0, 4 and 8 hours post-exposure. Cells were washed in cold PBS (without Ca^++^/Mg^++^). Cells were combined with molten agarose at 37°C and 50 ul was spread evenly across the circular sample area on the provided slides. Slides were placed at 4°C for 20 minutes then immersed in cold lysis buffer containing 10% DMSO overnight at 4°C. The next day, slides were immersed in neutral electrophoresis buffer for 30 minutes then aligned in an electrophoresis tank at 4°C, equidistance from the electrodes, filled with neutral electrophoresis buffer no more than 0.6 cm above the slides and 14 volts were applied for 45 minutes. Slides were then immersed in DNA precipitation buffer for 30 minutes at room temperature, followed be 70% ethanol for 30 minutes then dried at 37°C for at least 15 minutes. Slides were stained with DAPI and visualized under a 20x objective by epifluorescence microscopy using an Olympus BX50 Microscope with QImaging Retiga 2000R digital camera and software. Data analysis was performed using OpenComet software plugin for ImageJ 1.45s (NIH, USA). At least 50 nuclei were counted per experimental group.

### Cell cycle analysis

Treated and untreated cells were fixed in 1% paraformaldehyde in PBS with 5mM EDTA on ice. Cells were then permeabilized in 70% ethanol for at least 30 minutes at -20°C then rehydrated in 1% BSA/0.1% Triton X-100 in PBS for 30 minutes at 4°C. Rehydrated cells were resuspended in propidium iodide (PI)/RNase staining buffer (BD pharmingen) and filtered through a 44μM pore filter. Labeled cells were analyzed on a MACS VYB flow cytometer using 535nm excitation and PI fluorescence detection at 617nm. Percentages of cells in G0/G1, S, and G2/M were calculated using ModFit LT Version 4.1.7 (Verity Software House, Topsham ME).

### Invasion assay

The cell invasion assay (Millipore) was performed as per the manufacturer’s instructions. Briefly, the ECM layer of the insert was rehydrated and placed in the wells containing DMEM 10% FBS. MCF-7 cells expressing RAD51 WT or G151D were plated at 5x10^5^ cells per insert in serum-free DMEM. 48 hours after plating, cells that had not invaded were removed using a cotton-tipped swab. Cells that had invaded to the bottom of the transwell were stained in cell stain solution then washed in water. The relative invasiveness of the cells was measured by dissolving the stained cells in 10% acetic acid and performing a colometric reading (OD) at 560nm.

### DNA strand exchange assay

All DNA substrates were obtained PAGE purified from IDT. The following oligonucleotides were utilized: RJ-167-mer (5’-CTG CTT TAT CAA GAT AAT TTT TCG ACT CAT CAG AAA TAT CCG TTT CCT ATA TTT ATT CCT ATT ATG TTT TAT TCA TTT ACT TAT TCT TTA TGT TCA TTT TTT ATA TCC TTT ACT TTA TTT TCT CTG TTT ATT CAT TTA CTT ATT TTG TAT TA TCC TTA TCT TAT TTA-3’), RJ-PHIX-42-1 (5’-CGG ATA TTT CTG ATG AGT CGA AAA ATT ATC TTG ATA AAG CAG-3’), RJ-Oligo1 (5’-TAA TAC AAA ATA AGT AAA TGA ATA AAC AGA GAA AAT AAA G-3’), and RJ-Oligo2 (5’-CTT TAT TTT CTC TGT TTA TTC ATT TAC TTA TTT TGT ATT A-3’). The 3’ tail DNA substrate was generated by annealing RJ-167-mer to RJ-PHIX-42-1 at a 1:1 molar ratio. The dsDNA donor was generated by first radiolabeling RJ-Oligo1 with ^32^P (T4 Polynucleotide Kinase) on the 5’-end and annealing it to RJ-Oligo2 at a 1:1 molar ratio. The assay buffer contained: 25 mM TrisOAc (pH 7.5), 1 mM MgCl_2_, 2 mM CaCl_2_, 0.1 μg/μL BSA, 2 mM ATP, and 1 mM DTT. All pre-incubations and reactions were at 37°C. The protein and DNA substrates were used at the following concentrations: RAD51 (0.3 μM), 3’ tail DNA (4 nM molecules) and dsDNA (4 nM molecules). RPA and BRCA2 proteins were used at the concentrations indicated in the figure (unless otherwise noted). The 3’ tail DNA was incubated first with RPA for 5 minutes, followed by the addition of RAD51 for 5 minutes (or BRCA2 and RAD51 where indicated), and finally, the radiolabeled donor dsDNA was added for 30 minutes. Where proteins were omitted, storage buffer was substituted. In the case where RAD51 protein was titrated, the RPA protein was omitted. The reaction was terminated with Proteinase K/0.5% SDS for 10 minutes. The reactions were loaded on a 6% polyacrylamide gel in TAE buffer and electrophoresis was at 60 V for 80 minutes. The gel was then dried onto DE81 paper and exposed to a PhosphorImager screen overnight. The screen was scanned on a Molecular Dynamics Storm 840 PhosphorImager and bands quantified using ImageQuant software. The percentage of DNA strand exchange product was calculated as labeled product divided by total labeled input DNA in each lane.

### Single molecule fluorescence resonance energy transfer (smFRET)

All reactions were performed at room temperature in a buffer composed of 50 mM Tris-HCL, pH 8.0, 1 mM MgCl 2, 2 mM CaCl 2, 0.1 mg/mL BSA and an oxygen scavenging system (1 mg/ml glucose oxidase, 0.4% (w/v) D-glucose, 0.02 mg/ml catalase, and 2 mM Trolox) [1]. smFRET assays were preformed according to previously described protocols [77,78]. In brief, 50 pM– 300 pM DNA (Supplementary Table 1) was tethered to a PEG-coated quartz surface through biotin-neutravidin linkage followed by the addition of proteins. Data were recorded and analyzed by scripts written in IDL, which extracted corresponding single-molecule donor and acceptor spots into single-molecule trajectories. FRET efficiency (E_FRET_) was calculated as the ratio between the acceptor intensity and the sum of the acceptor and donor intensities. Programs written in Matlab were used to view and analyze FRET trajectories. Histograms were generated using a sample size of over 75 individual molecular trajectories.

### DNA fiber assay

Cells were grown in appropriate media until 30–40% confluent. Cells were pulsed with 25 μM (final concentration) of 5-Iodo-2’-deoxyuridine (IdU) (Sigma) for 20 minutes at 37°C. Cells were washed with PBS then treated with 0.5 mM hydroxyurea (HU) (Sigma) for 2hrs, exposed to 8GY IR or untreated. Cells were then pulsed with 250 μM (final concentration) of 5-Chloro-2’-deoxy-uridine (CIdU) for 20 minutes at 37°C. Cells were harvested, spotted onto microscope slides then lysed in fiber lysis solution (50 mM EDTA, 0.5% SDS, 200 mM Tris-HCl, pH 7.5 in ddH_2_O) for 2 minutes. Slides were then tilted at a 15° angle to allow DNA fibers to spread on slide then allowed to air-dry. Slides were fixed in 75% methanol/25% acetic acid then placed in 2.5 M HCl for 3 hours. Slides were washed in ddH_2_O then blocked in 5% BSA. IdU-incorporated replication tracts were labeled using a mouse anti-BrdU primary antibody (1/400) (BD Biosciences) and CIdU-incorporated replication tracts were labeled using rat anti-BrdU primary antibody (1:25) (Abcam) for 2 hrs at RT. Slides were washed then labeled with goat anti-rat Alexa Flour 488 (Invitrogen) secondary antibody (1/200) and goat anti-mouse Texas Red (Santa Cruz) secondary antibody (1/200) for 2.5 hrs at RT. Slides were mounted in Prolong Gold antifade mounting media (Invitrogen) and visualized under a 60x objective by epifluorescence microscopy using an Olympus BX50 Microscope with QImaging Retiga 2000R digital camera and software. Data analysis was performed using ImageJ v. 2.0.0 (NIH, USA). At least 100 elongating replication structures were measured per cell line/per experimental group for replication tract length. The data are representative of 3 independent experiments. ***p<0.001; **p<0.01.

### Affinity protein pull-downs

Amylose pull-down assays were performed by transiently transfecting 1 μg of the indicated constructs into 5x10^5^ 293TD cells/well seeded in 6-well plates using TurboFect (Thermo Fisher Scientific). 36 hours post-transfection, the cells were harvested in 500 μL of buffer ‘BB’: (50 mM HEPES [pH = 7.5], 250 mM NaCl, 1% Igepal CA-630, 1 mM MgCl_2_, 1 mM DTT, 250 Units/mL Benzonase (EMD Millipore), and 1X EDTA-free protease inhibitor cocktail (Roche). Cell lysates were batch bound to 20 μL of amylose resin for 2 hours. The bound proteins were washed two times with buffer ‘B’: 50 mM HEPES (pH 7.5), 0.5% Igepal CA-630, 0.1% Triton X-100, 0.5 mM EDTA, and 1 mM DTT containing 1M NaCl followed by two washes in buffer B containing 250 mM NaCl. Purified RAD51 at the concentrations indicated in the figure were then added and incubated at 37°C for 30 minutes. The protein complexes were then washed again three times with buffer B containing 250 mM NaCl followed by elution in 20 μL of 10 mM maltose. Loading sample buffer was added, samples were heated at 54°C for 4 minutes, and loaded onto a 4–15% gradient SDS-polyacrylamide gel (Bio-Rad TGX Stain-Free gel). The gel was run for 2 hours at 100 Volts. The proteins were visualized by staining with SyproOrange (Invitrogen) and quantified using ImageQuant software on a Storm 860 PhosphorImager (Molecular Dynamics). RAD51 non-specific binding to amylose beads was negligible.

### Electrophoretic mobility shift assay

Oligonucleotide substrates were obtained PAGE purified from IDT. To generate the 3’ tail DNA substrate, RJ-167-mer was radiolabeled with ^32^P at the 5’-end using T4 Polynucleotide Kinase (NEB) and then annealed at a 1:1 molar ratio to RJ-PHIX-42-1. ssDNA was the radiolabeled RJ-167-mer alone and the dsDNA substrate was RJ-Oligo1 labeled at the 5’-end by ^32^P (T4 Polynucleotide Kinase) and annealed to RJ-Oligo2 at 1:1 molar ratio. RAD51 (at the indicated concentrations) was incubated with 0.2 nM (molecules) of the radiolabeled DNA substrate for 30 min at 37°C in DNA strand exchange buffer (25 mM TrisOAc [pH 7.5], 1 mM MgCl_2_, 2 mM CaCl_2_, 0.1 μg/μL BSA, 2 mM ATP, and 1 mM DTT). The reactions were resolved by electrophoresis on a 6% polyacrylamide gel in TAE (40 mM Tris-acetate [pH 7.5], 0.5 mM EDTA) buffer for 90 minutes at 80 V in the cold room (4°C). The gel was then dried onto DE81 paper and exposed to a PhosphorImager screen overnight. The screen was scanned on a Molecular Dynamics Storm 840 PhosphorImager and bands quantified using ImageQuant software. The percentage of protein-DNA complexes was calculated as the free radiolabeled DNA remaining in a given lane relative to the protein-free lane, which defined the value of 0% complex (100% free DNA).

## Supporting Information

S1 Fig Enhanced HDR of chromosomal DSBs in U2OS-DRGFP cell lines expressing RAD51 G151D. **A.** Western blot demonstrates equivalent expression of exogenous (I) RAD51 WT and G151D, as well as the fold increase in expression over endogenous RAD51 (I/R), in their respective U2OS-DRGFP pools. **B.** Percentage of GFP positive cells was measured by flow cytometry as described in Fig 2B. Data are graphed as mean ± SD from 3 independent experiments. * p<0.05.
(TIF)

S2 Fig The impact of RAD51 WT or G151D expression on cell cycle. **A,B.** MCF10A pools expressing RAD51 WT or G151D were exposed to 8GY ionizing radiation then fixed/permeabilized at 0, 2, 4, 8 post-IR exposure **(A)** or untreated cells **(B)** were fixed/permeabilized and both populations were labeled with propidium iodide to measure DNA content by flow cytometry. The percentage of cells in S and G2/M phases of the cell cycle were scored using ModFit analysis. The data are graphed as mean ± SD of 2 independent experiments. **p<0.01; ***p<0.001.
(TIF)

S3 Fig Endogenous levels of DNA damage in MCF10A cells expressing RAD51 WT or G151D. **A,B.** MCF10A pools expressing RAD51 WT or G151D were harvested and single cell electrophoresis was performed to quantitate DNA damage using the comet assay. **A.** Data are graphed as mean ± SEM. **B.** Representative images from RAD51 WT or G151D expressing cells. **C,D.** MCF10A pools expressing WT or G151D were labeled with a γH2AX antibody (green) then mounted in Prolong Gold mounting media containing DAPI (blue, nuclei). Labeled cells were visualized using a Zeiss LSM 510 META confocal imaging system. **C.** The number of nuclei with γH2AX was counted. The data are graphed as mean ± SEM (n>500 nuclei) **** p< 0.0001; *** p<0.001. **D.** Representative images of γH2AX foci in MCF10A RAD51 WT and RAD51 G151D expressing pools.
(TIF)

S4 Fig Increased resistance to IR in MCF-7 cells expressing RAD51 G151D. Serially diluted RAD51 WT and RAD51 G151D expressing MCF-7 pools were X-irradiated at 0, 2, 4, or 8 GY. After 10 days, colonies were stained with crystal violet and scored. Data are representative of 3 independent experiments and graphed as mean ± SD.
(TIF)

S5 Fig Amylose affinity pull-downs of either 2XMBP-BRCA2 or 2XMBP-PALB2 expressed in human 293T cells and incubated with RAD51 WT or G151D. **A.** SyproOrange stained SDS-PAGE gel depicting either BRCA2 (left) or PALB2 (right) incubated with increasing concentrations of purified WT RAD51 or G151D. **B.** RAD51 WT and G151D binding to BRCA2 (left graph) or PALB2 (right graph) was quantitated using ImageQuant software and normalized to the band intensities of BRCA2 and PALB2 respectively. This experiment was repeated twice. A representative gel image is shown.
(TIF)

S6 Fig **A.** Schematic of DNA strand exchange assay utilizing RAD51 protein only. **B.** Autoradiograms of DNA strand exchange in the presence of increasing concentrations of RAD51 WT (upper gel) or G151D (lower gel). Lanes 1 and 11 are no protein controls. **C.** Quantification of the gels shown in **(B)**. Error bars are S.D., (n = 3).
(TIF)

S7 Fig EMSA (electrophoretic mobility shift assay) analysis of RAD51 WT and G151D. **A.** Autoradiograms of increasing concentrations of RAD51 WT and G151D incubated with 3’ tail DNA, ssDNA, and dsDNA radiolabeled substrates. G151D protein-DNA complexes resolve at a faster mobility than WT RAD51. **B.** Quantification of the gels shown in **(A)** depicting the percentage of RAD51-DNA complexes.
(TIF)
