# Supplementary figures and images for: The Tumor-Associated Variant RAD51 G151D Induces a Hyper-Recombination Phenotype
Source: PLoS Genet. 2016 Aug 11;12(8):e1006208. doi: 10.1371/journal.pgen.1006208 (PMC4981402; doi:10.1371/journal.pgen.1006208)

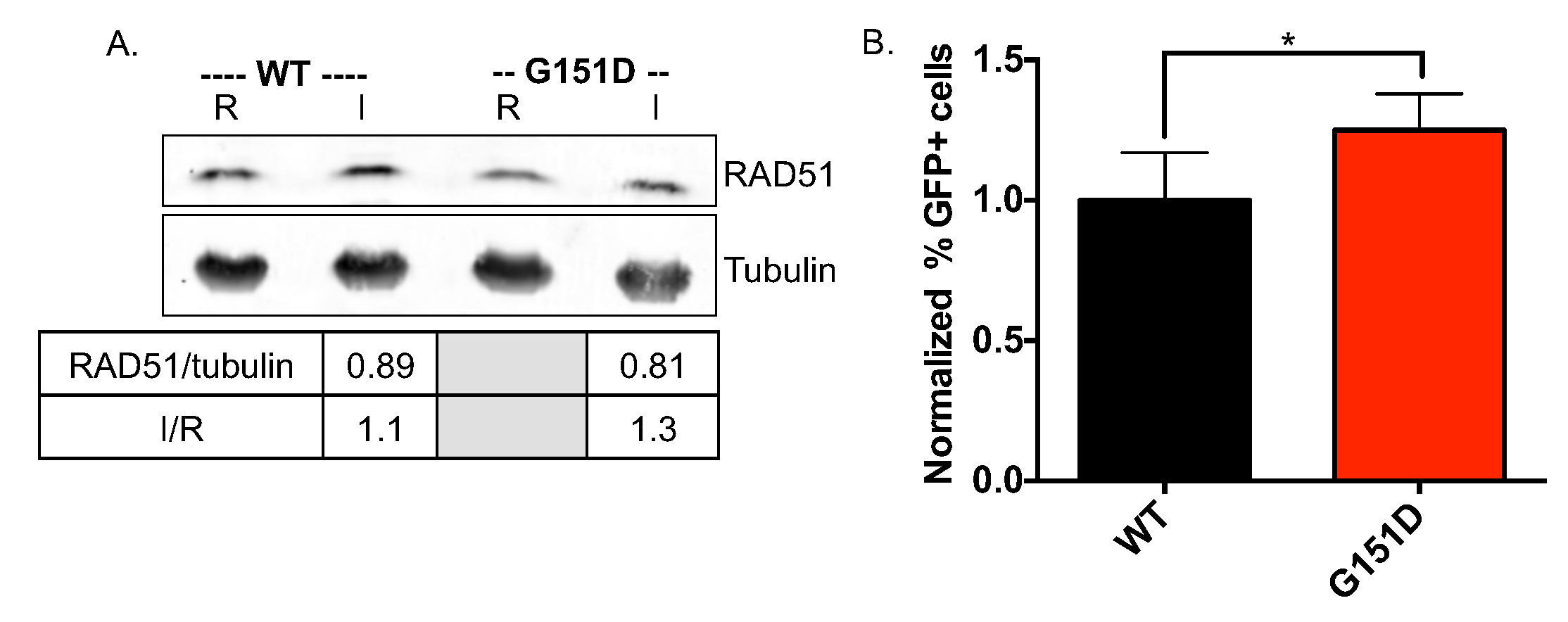

Supplement: S1 Fig — A. Western blot demonstrates equivalent expression of exogenous (I) RAD51 WT and G151D, as well as the fold increase in expression over endogenous RAD51 (I/R), in their respective U2OS-DRGFP pools. B. Percentage of GFP positive cells was measured by flow cytometry as described in Fig 2B. Data are graphed as mean ± SD from 3 independent experiments. * p<0.05. (TIF) [file pgen.1006208.s001.tif]

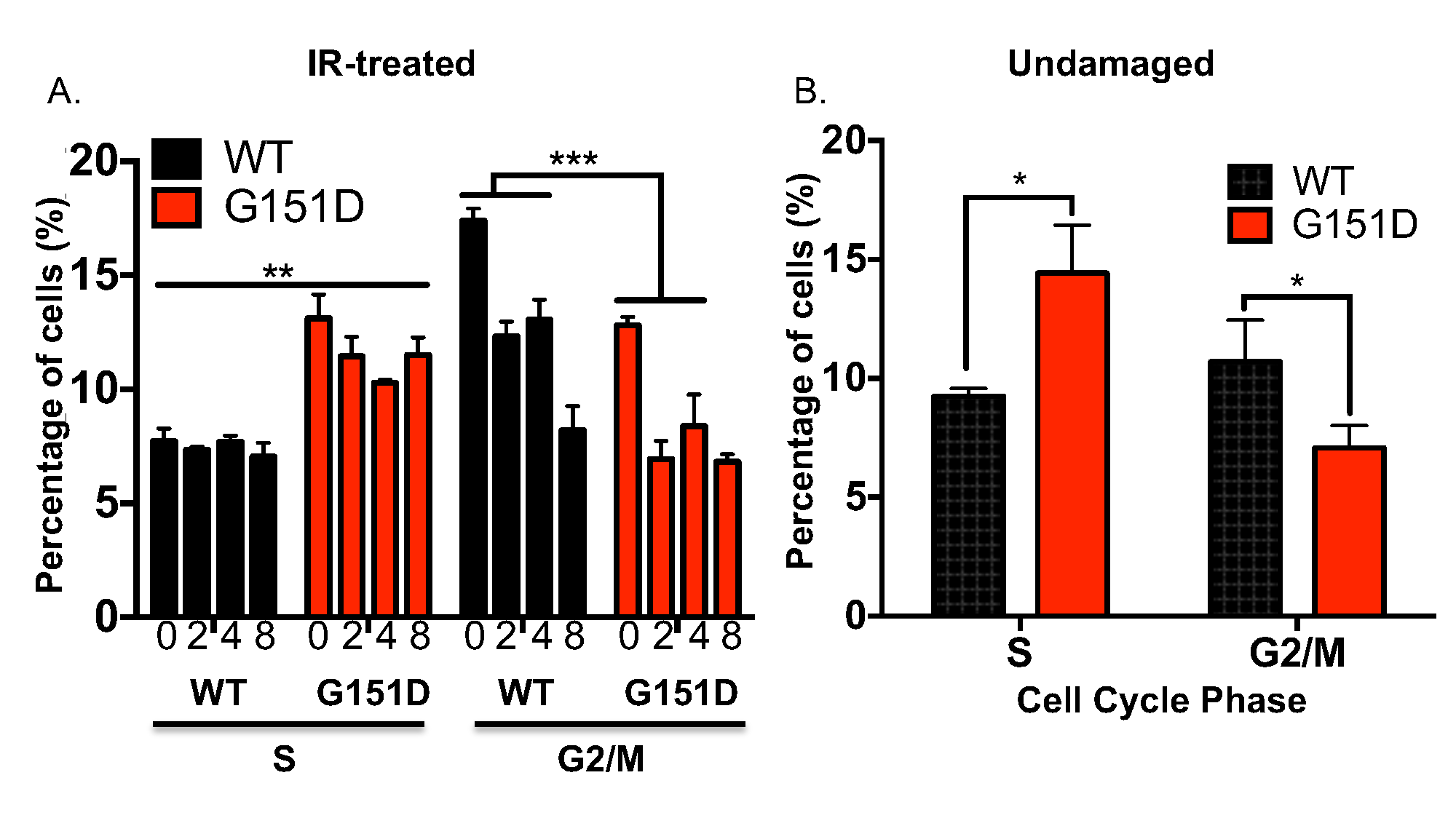

Supplement: S2 Fig — A,B. MCF10A pools expressing RAD51 WT or G151D were exposed to 8GY ionizing radiation then fixed/permeabilized at 0, 2, 4, 8 post-IR exposure (A) or untreated cells (B) were fixed/permeabilized and both populations were labeled with propidium iodide to measure DNA content by flow cytometry. The percentage of cells in S and G2/M phases of the cell cycle were scored using ModFit analysis. The data are graphed as mean ± SD of 2 independent experiments. **p<0.01; ***p<0.001. (TIF) [file pgen.1006208.s002.tif]

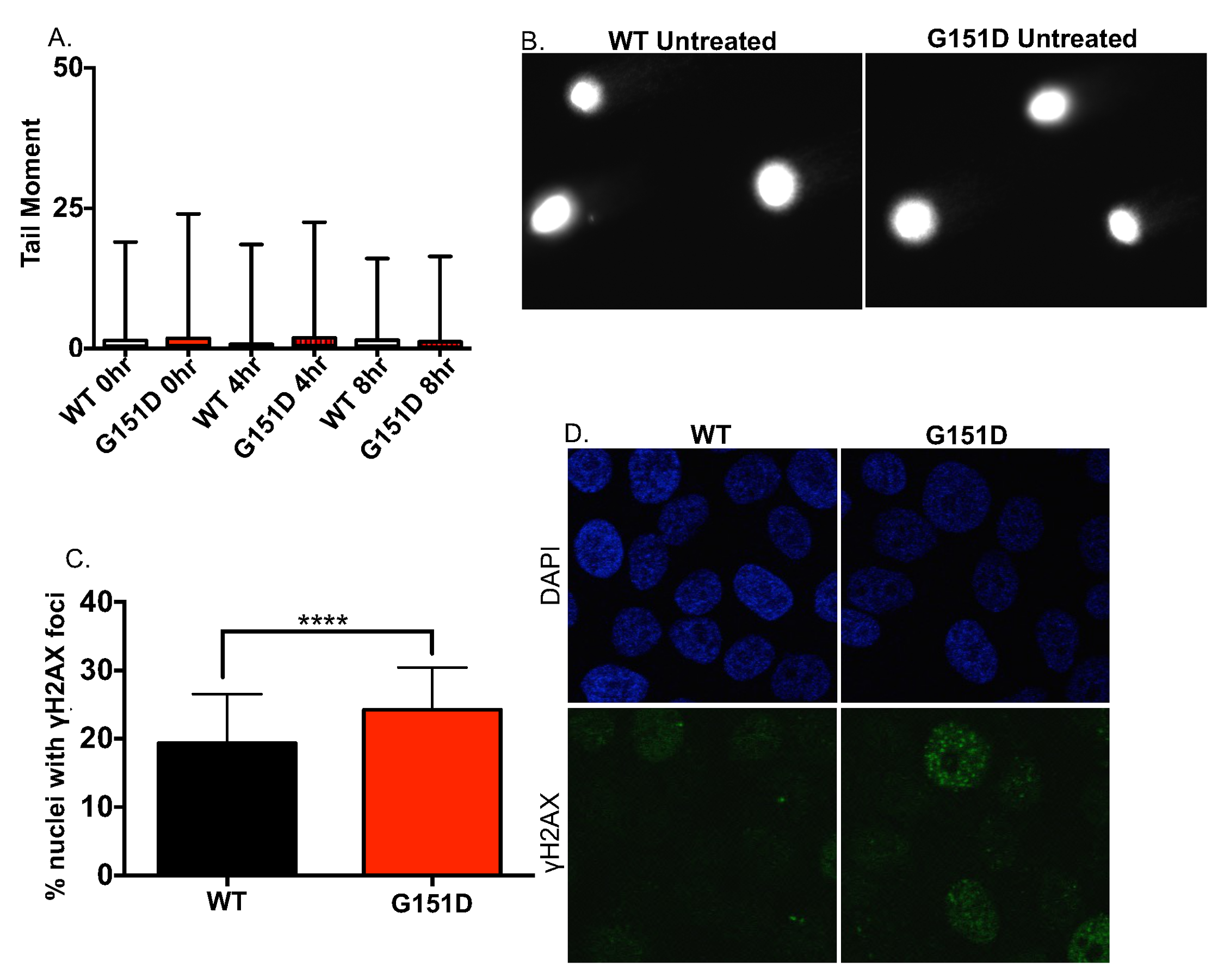

Supplement: S3 Fig — A,B. MCF10A pools expressing RAD51 WT or G151D were harvested and single cell electrophoresis was performed to quantitate DNA damage using the comet assay. A. Data are graphed as mean ± SEM. B. Representative images from RAD51 WT or G151D expressing cells. C,D. MCF10A pools expressing WT or G151D were labeled with a γH2AX antibody (green) then mounted in Prolong Gold mounting media containing DAPI (blue, nuclei). Labeled cells were visualized using a Zeiss LSM 510 META confocal imaging system. C. The number of nuclei with γH2AX was counted. The data are graphed as mean ± SEM (n>500 nuclei) **** p< 0.0001; *** p<0.001. D. Representative images of γH2AX foci in MCF10A RAD51 WT and RAD51 G151D expressing pools. (TIF) [file pgen.1006208.s003.tif]

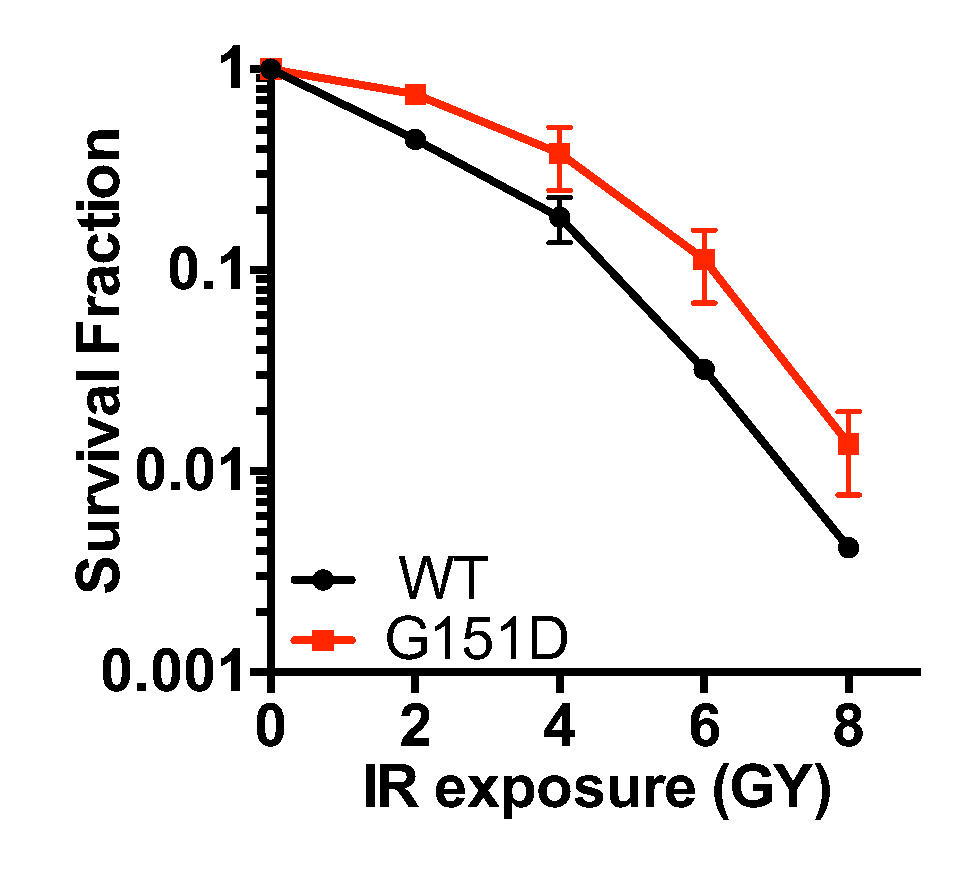

Supplement: S4 Fig — Serially diluted RAD51 WT and RAD51 G151D expressing MCF-7 pools were X-irradiated at 0, 2, 4, or 8 GY. After 10 days, colonies were stained with crystal violet and scored. Data are representative of 3 independent experiments and graphed as mean ± SD. (TIF) [file pgen.1006208.s004.tif]

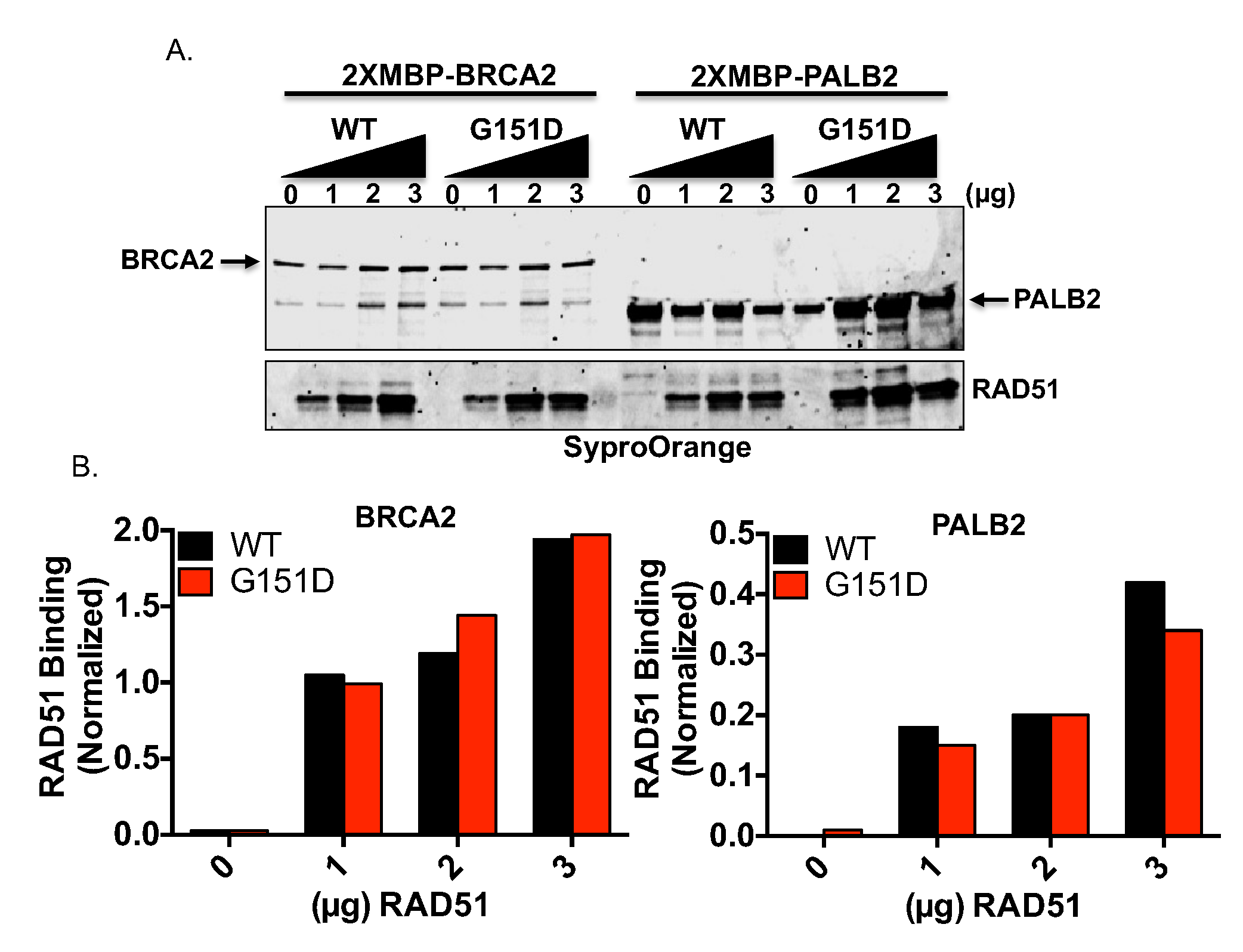

Supplement: S5 Fig — A. SyproOrange stained SDS-PAGE gel depicting either BRCA2 (left) or PALB2 (right) incubated with increasing concentrations of purified WT RAD51 or G151D. B. RAD51 WT and G151D binding to BRCA2 (left graph) or PALB2 (right graph) was quantitated using ImageQuant software and normalized to the band intensities of BRCA2 and PALB2 respectively. This experiment was repeated twice. A representative gel image is shown. (TIF) [file pgen.1006208.s005.tif]

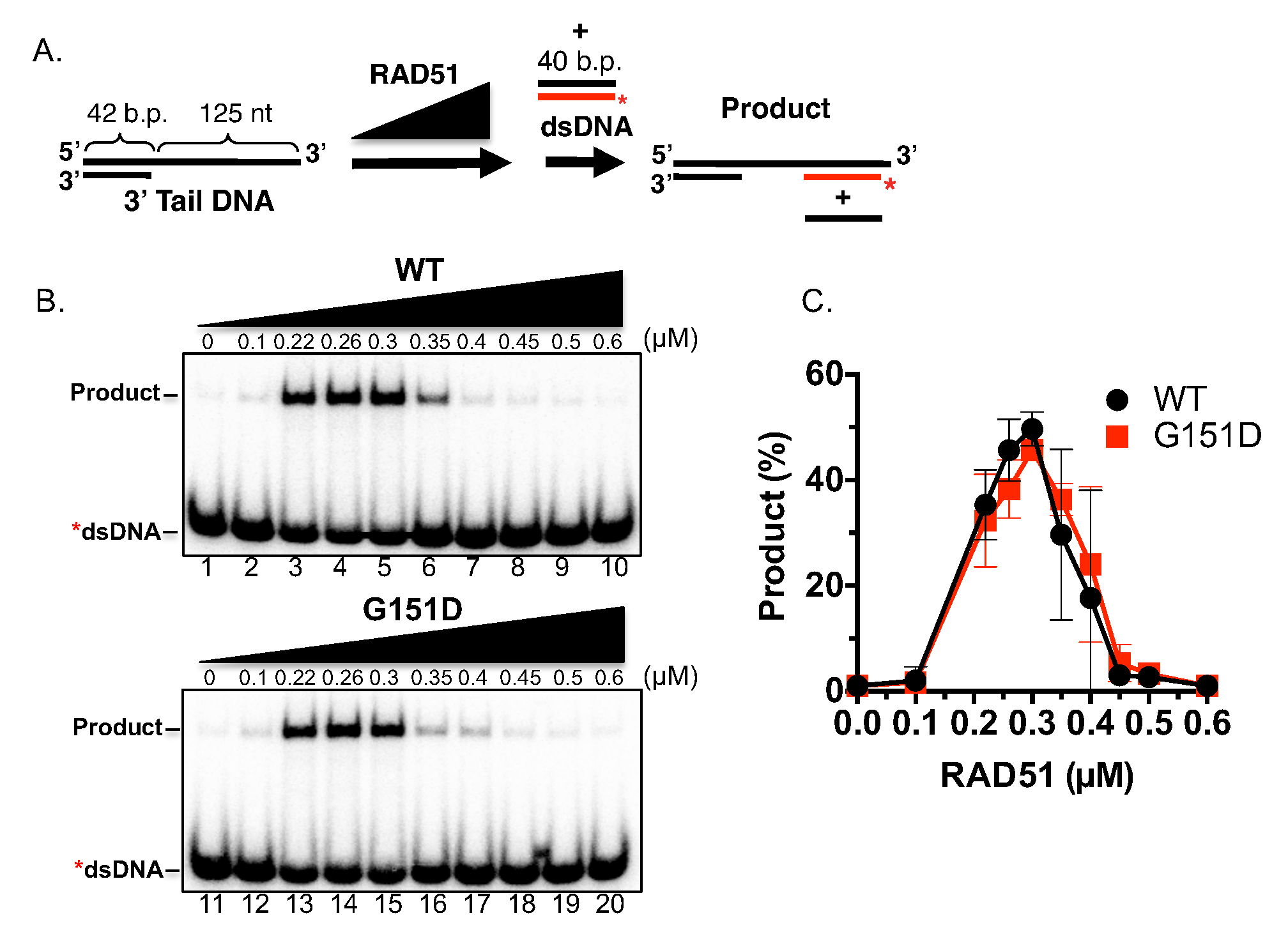

Supplement: S6 Fig — A. Schematic of DNA strand exchange assay utilizing RAD51 protein only. B. Autoradiograms of DNA strand exchange in the presence of increasing concentrations of RAD51 WT (upper gel) or G151D (lower gel). Lanes 1 and 11 are no protein controls. C. Quantification of the gels shown in (B). Error bars are S.D., (n = 3). (TIF) [file pgen.1006208.s006.tif]

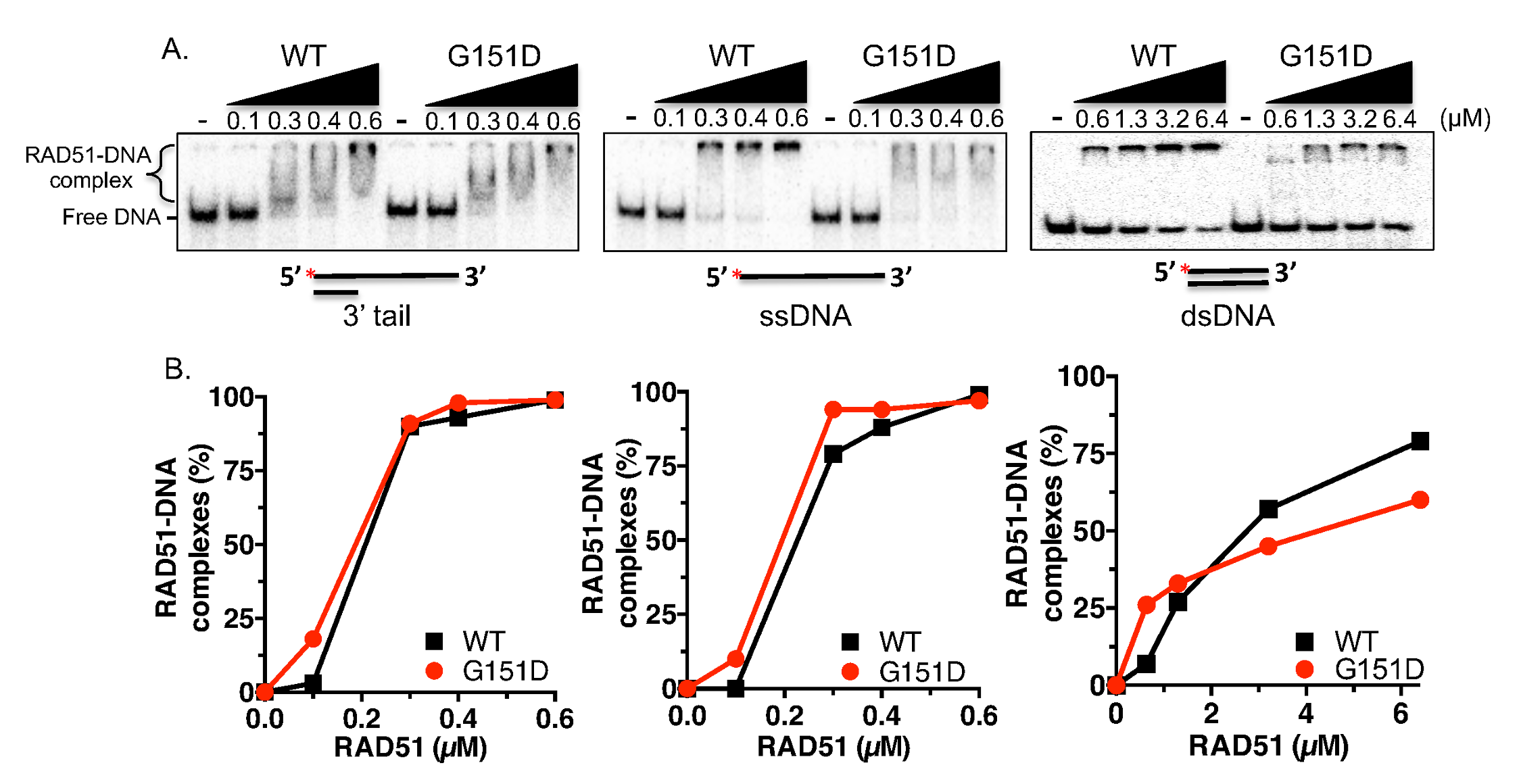

Supplement: S7 Fig — A. Autoradiograms of increasing concentrations of RAD51 WT and G151D incubated with 3’ tail DNA, ssDNA, and dsDNA radiolabeled substrates. G151D protein-DNA complexes resolve at a faster mobility than WT RAD51. B. Quantification of the gels shown in (A) depicting the percentage of RAD51-DNA complexes. (TIF) [file pgen.1006208.s007.tif]
